# Supplementary material for: Harnessing peak transmission around symptom onset for non-pharmaceutical intervention and containment of the COVID-19 pandemic
Source: Nat Commun. 2021 Feb 19;12:1147. doi: 10.1038/s41467-021-21385-z (PMC7895830; doi:10.1038/s41467-021-21385-z)
Supplement: Supplementary file 1 — Supplementary information [file 41467_2021_21385_MOESM1_ESM.pdf]

Supplementary Information

# Harnessing peak transmission around symptom onset for non-pharmaceutical intervention and containment of the COVID-19 pandemic

Liang Tian, Xuefei Li, Fei Qi, Qian-Yuan Tang, Viola Tang, Jiang Liu, Zhiyuan Li, Xingye Cheng, Xuanxuan Li, Yingchen Shi, Haiguang Liu, Lei-Han Tang

In this Supplementary Information, a stochastic model of COVID-19 transmission in a homogeneous population is presented and analysed. Disease progression of an individual is compartmentalised into latent (L), pre-symptomatic infectious (A) and symptomatic (S) phases. To better accommodate the transmission characteristics of COVID-19 at the population level, the A phase is further split into two sub-periods, $A_1$ before the infectiousness peak with a variable duration, and $A_2$ after the infectiousness peak with a fixed duration. With regard to the transmission capacity, the $A_2$ phase and the S phase are treated as a single stretch of declining infectiousness. For a sufficiently large population, the size of infected subpopulations satisfies an integral renewal equation. We derive the kernel function from the model and calibrate it against clinical case studies. Analytical and numerical solutions of the integral equation with and without intervention are presented.

From a mathematical point of view, our model can be considered as a special case of the general class of epidemic models originally introduced by Kermack and McKendrick [1] and expanded over the years. For a historical review and modern treatment, we refer the reader to Refs. [2, 3, 4]. We would like to thank the anonymous reviewers for bringing these classical works to our attention.

## Contents

1 The Transmission Model 3
1.1 The model and governing equations 3
1.2 Disease progression and symptom onset time 5
1.3 Exponential growth/decay 6
1.4 Percentage of subpopulations during exponential growth 7

2 Model Calibration against Case Studies 8
2.1 Symptom onset time distribution 8
2.1.1 Hybrid log-normal distribution with an exponential tail 8
2.1.2 Hybrid Weibull distribution with an exponential tail 9
2.1.3 Uncertainty analysis through bootstrap re-sampling 10
2.2 Transmission characteristics 10
2.2.1 Infectiousness around symptom onset 10
2.2.2 A conglomerate of two exponential wings 11
2.2.3 Exponential wings with a flat cap 12
2.2.4 Exponential wings with a smooth cap 12
2.2.5 Uncertainty analysis through bootstrap re-sampling 13

2.3 Serial interval statistics . . . . . 13
2.4 Non-Markovian transition rate from latent to infectious phase . . . . . 14

3 Simplifying Approximations 15
3.1 An approximate formula for the mean reproduction rate . . . . . 15
3.2 A Markov model . . . . . 16

4 Model Exploration under Intervention 18
4.1 Quarantine of infected individuals . . . . . 18
4.1.1 Mean reproduction number from day $t$ onward since infection . . . . . 18
4.1.2 Testing and quarantine . . . . . 18
4.1.3 Contact tracing . . . . . 19
4.2 Mask wearing . . . . . 19
4.3 Solution with imported cases . . . . . 21
4.4 Crossover behaviour under linear transmission reduction . . . . . 22
4.5 Homestay . . . . . 23

5 Estimation of exponential growth rates during initial outbreaks 25

References 28

# 1 The Transmission Model

## 1.1 The model and governing equations

The basic structure of our model follows Main Text Figure 1, with model parameters defined in Supplementary Figure 1. The disease progression parameters $\alpha_{\rm A}$ and $\alpha_{\rm L}(t_{\rm L})$ are taken to be universal, while the transmission rates $\beta_{\rm A}$ and $\beta_{\rm B}(t_{\rm B})$ may vary significantly from community to community.

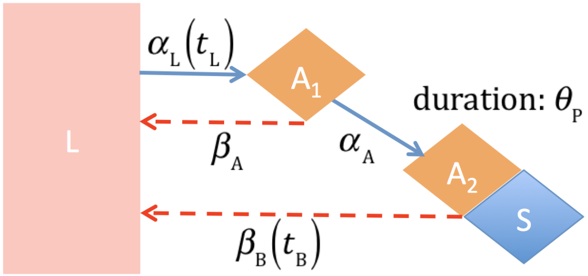


**Supplementary Figure 1: A stochastic model for disease progression and transmission**. Disease progression of an infected individual is assumed to be described by a renewal process following the sequence of latent (L), pre-symptomatic infectious ($\rm A_1$ and $\rm A_2$) and symptomatic (S) phases. The transition rate from L to $\rm A_1$ is given by $\alpha_{\rm L}(t_{\rm L})$ which depends on the dwell time $t_{\rm L}$ in the latent phase. The transition from $\rm A_1$ to $\rm A_2$, which coincides with peak infectiousness, is Poisson at a constant rate $\alpha_{\rm A}$. The $\rm A_2$ phase has a fixed duration $\theta_{\rm P}$, after which the patient enters S phase. All three phases $\rm A_1$, $\rm A_2$ and S are infectious, with transmission rates to reproduce secondary cases given by $\beta_{\rm A}$ ($\rm A_1$ phase) and $\beta_{\rm B}(t_{\rm B})$ ($\rm A_2$ and S phases combined), respectively. The latter is a function of $t_{\rm B}$, the number of days since the start of the $\rm A_2$ phase.

We now consider groups of infected individuals in L, $\rm A_1$, $\rm A_2$ and S in a large and well-mixed population, using italic symbols to denote their size. We shall also assume that the number of individuals in each of the disease phases is sufficiently large, so that stochasticity arising from the random transition events to enter the next phase is averaged out, leading to deterministic equations at the population level. Our experience with numerical simulations of the stochastic model indicates that ten is already a good size for the subpopulations for this purpose. Unless otherwise specified, we will use the capital letter $T$ to denote calendar time, and the lower case letters $t, t_{\rm A}, t_{\rm B}$ to denote elapsed times since infection and upon entering the $\rm A_1$ and $\rm A_2$ phases, respectively.

In a closed community, the number of new infections per unit time (also known as "incidence") at time $T$ is given by,

$$J_{\rm L}(T) = \beta_{\rm A} A_1(T) + \int_0^\infty \beta_{\rm B}\left(t_{\rm B}\right)\alpha_{\rm A} A_1\left(T - t_{\rm B}\right) dt_{\rm B}. \tag{S1}$$

The first term is due to transmission by infected individuals in the $\rm A_1$ phase. The second term is due to individuals who have transited to the post $\rm A_1$ phase at an earlier time $T - t_{\rm B}$. Each newly infected individual then follows the disease progression path shown by the blue arrows in Supplementary Figure 1, with the transition rates as specified. In particular, the mean flux to $\rm A_1$ at time $T$ due to a group infected in the time interval $(T - t, T - t + dt)$ is given by,

$$dJ_{\rm A_1}(T) = \alpha_{\rm L}(t) q_{\rm L}(t) J_{\rm L}(T - t) dt,$$

where

$$q_{\rm L}(t) = e^{-\int_0^t \alpha_{\rm L}(t_1) dt_1}$$

is the probability that an individual remains in the latent phase L on day $t$ since infection. Adding up contributions from all such groups, we obtain

$$J_{\mathrm{A}_1}(T) = \int_0^\infty \alpha_{\mathrm{L}}(t) q_{\mathrm{L}}(t) J_{\mathrm{L}}(T-t) dt. \tag{S2}$$

Given that the exit rate from the $\mathrm{A}_1$ phase is given by $\alpha_{\mathrm{A}}$, we may write,

$$\begin{aligned} A_1(T) &= \int_0^\infty e^{-\alpha_{\mathrm{A}} t_{\mathrm{A}}} J_{\mathrm{A}_1}(T-t_{\mathrm{A}}) dt_{\mathrm{A}} \\ &= \int_0^\infty e^{-\alpha_{\mathrm{A}} t_{\mathrm{A}}} \Big[ \int_0^\infty \alpha_{\mathrm{L}}(t_1) q_{\mathrm{L}}(t_1) J_{\mathrm{L}}(T-t_{\mathrm{A}}-t_1) dt_1 \Big] dt_{\mathrm{A}} \\ &= \int_0^\infty \Big[ \int_0^t \alpha_{\mathrm{L}}(t_1) q_{\mathrm{L}}(t_1) e^{-\alpha_{\mathrm{A}}(t-t_1)} dt_1 \Big] J_{\mathrm{L}}(T-t) dt. \end{aligned} \tag{S3}$$

The last line is obtained with the help of the identity,

$$\int_0^\infty dx \int_0^\infty dy f(x) g(y) h(x+y) = \int_0^\infty \Big[ \int_0^z f(z-y) g(y) dy \Big] h(z) dz. \tag{S4}$$

Equations (S1) and (S3) allow us to obtain the following closed equation,

$$J_{\mathrm{L}}(T) = \int_0^\infty r(t) J_{\mathrm{L}}(T-t) dt. \tag{S5}$$

Here

$$r(t) = \int_0^t \alpha_{\mathrm{L}}(t_1) q_{\mathrm{L}}(t_1) e^{-\alpha_{\mathrm{A}}(t-t_1)} \Big[ \beta_{\mathrm{A}} + \int_0^{t-t_1} \beta_{\mathrm{B}}(t_{\mathrm{B}}) e^{\alpha_{\mathrm{A}} t_{\mathrm{B}}} \alpha_{\mathrm{A}} dt_{\mathrm{B}} \Big] dt_1 \tag{S6}$$

is the mean reproduction rate of a viral carrier in day $t$ since infection. The first term on the right-hand side of (S6) is quite obvious. To obtain the second term, we write the term inside the square brackets of Eq. (S3) as,

$$w(t) = \int_0^t \alpha_{\mathrm{L}}(t_1) q_{\mathrm{L}}(t_1) e^{-\alpha_{\mathrm{A}}(t-t_1)} dt_1.$$

Consequently,

$$\begin{aligned} & \int_0^\infty \beta_{\mathrm{B}}(t_{\mathrm{B}}) \alpha_{\mathrm{A}} A_1(T-t_{\mathrm{B}}) dt_{\mathrm{B}} \\ = & \int_0^\infty \beta_{\mathrm{B}}(t_{\mathrm{B}}) \alpha_{\mathrm{A}} \Big[ \int_0^\infty w(t) J_{\mathrm{L}}(T-t_{\mathrm{B}}-t) dt \Big] dt_{\mathrm{B}} \\ = & \int_0^\infty \Big[ \int_0^t \beta_{\mathrm{B}}(t_{\mathrm{B}}) \alpha_{\mathrm{A}} w(t-t_{\mathrm{B}}) dt_{\mathrm{B}} \Big] J_{\mathrm{L}}(T-t) dt, \end{aligned}$$

where we have used Eq. (S4) for the last step. Furthermore,

$$\begin{aligned} & \int_0^t \beta_{\mathrm{B}}(t_{\mathrm{B}}) \alpha_{\mathrm{A}} w(t-t_{\mathrm{B}}) dt_{\mathrm{B}} \\ = & \int_0^t \beta_{\mathrm{B}}(t_{\mathrm{B}}) \alpha_{\mathrm{A}} \Big[ \int_0^{t-t_{\mathrm{B}}} \alpha_{\mathrm{L}}(t_1) q_{\mathrm{L}}(t_1) e^{-\alpha_{\mathrm{A}}(t-t_{\mathrm{B}}-t_1)} dt_1 \Big] dt_{\mathrm{B}} \\ = & \int_0^t \alpha_{\mathrm{L}}(t_1) q_{\mathrm{L}}(t_1) e^{-\alpha_{\mathrm{A}}(t-t_1)} \Big[ \int_0^{t-t_1} \beta_{\mathrm{B}}(t_{\mathrm{B}}) \alpha_{\mathrm{A}} e^{\alpha_{\mathrm{A}} t_{\mathrm{B}}} dt_{\mathrm{B}} \Big] dt_1 \end{aligned}$$

as required.

Equation (S5) implies that the temporal evolution of any linear function of $J_{\rm L}$ in the form

$$X(T)=\int_0^\infty a(t_1)J_{\rm L}(T-t_1)dt_1$$

satisfies the renewal equation

$$X(T)=\int_0^\infty r(t)X(T-t)dt. \tag{S7}$$

This can be shown easily as follows:

$$\begin{aligned} X(T) &= \int_0^\infty a(t_1)\Big[\int_0^\infty r(t)J_{\rm L}(T-t_1-t)dt\Big]dt_1 \\ &= \int_0^\infty r(t)\Big[\int_0^\infty a(t_1)J_{\rm L}(T-t_1-t)dt_1\Big]dt \\ &= \int_0^\infty r(t)X(T-t)dt. \end{aligned}$$

Equations (S5) and (S7), with the kernel function $r(t)$ given by (S6), constitute the fundamental dynamical equations for epidemic development at the population level. To include imported cases at the beginning or during an outbreak, a source term should be added to Eq. (S5), as we illustrate in Sec. 4.5.

## 1.2 Disease progression and symptom onset time

The symptom onset separates the $\rm A_2$ phase from the S phase. In our model, the $\rm A_2$ phase is assigned a fixed duration $\theta_{\rm P}$, the time lag between the peak infectiousness and the symptom onset. Consequently, we may express the symptom onset time as,

$$t_{\rm O}=t_{\rm L}+t_{\rm A1}+\theta_{\rm P},$$

where $t_{\rm L}$ and $t_{\rm A1}$ are the times an infected person spent in the latent and $\rm A_1$ phases, respectively. Since the statistics of $t_{\rm A1}$ follows a Markov process with a time constant $1/\alpha_A$ in our model, one may infer the statistics of $t_{\rm L}$ from the statistics of $t_{\rm O}$. This then allows us to determine the kernel function $r(t)$ and other quantities of interest in terms of the symptom onset time distribution $p_{\rm O}(t)$, which can be estimated from clinical data (see the section below). The function $p_{\rm O}(t)$ is also known as the incubation period distribution. We use the two terms interchangeably both in SI and in the Main Text.

In Supplementary Table 1 we give the probabilities for an individual to be in one of the four phases on day $t$ since infection. Expressions in the last two rows are quite obvious. The transition probability current from $\rm A_1$ to $\rm A_2$ at time $t$ is given by $\alpha_{\rm A}q_{\rm A_1}(t)$, which is the same as the probability density for symptom onset at a later time $t+\theta_{\rm P}$. This gives the expression for $q_{\rm A_1}(t)$ in Supplementary Table 1. The expression for $q_{\rm L}(t)$ is obtained simply by requiring that the sum of the probabilities is equal to one.

From the expression for $q_{\rm L}(t)$ in Supplementary Table 1, we obtain its Laplace transform,

$$\tilde{q}_{\rm L}(\lambda)=\frac{1}{\lambda}-\left(\frac{1}{\alpha_{\rm A}}+\frac{1}{\lambda}\right)e^{\lambda\theta_{\rm P}}\tilde{p}_{\rm O}(\lambda), \tag{S8}$$

where we have set $p_{\rm O}(t)=0$ for $t<\theta_{\rm P}$. The Laplace transform of $\alpha_{\rm L}(t)q_{\rm L}(t)=-\dot{q}_{\rm L}(t)$ is given by $1-\lambda\tilde{q}_{\rm L}(\lambda)$. Noting that Eq. (S6) is in the form of a convolution integral, we obtain,

$$\begin{aligned} \tilde{r}(\lambda) &= \Big(1-\lambda\tilde{q}_{\rm L}(\lambda)\Big)\left(\frac{\beta_{\rm A}}{\lambda+\alpha_{\rm A}}+\frac{\alpha_{\rm A}}{\lambda+\alpha_{\rm A}}\tilde{\beta}_{\rm B}(\lambda)\right) \\ &= \Big(\frac{\beta_{\rm A}}{\alpha_{\rm A}}+\tilde{\beta}_{\rm B}(\lambda)\Big)e^{\lambda\theta_{\rm P}}\tilde{p}_{\rm O}(\lambda). \end{aligned} \tag{S9}$$

**Supplementary Table 1: Probabilities for an individual to be in each of the disease phases on day $t$ since infection.**

| Phase | Probability | Expression |
|---|---|---|
| Latent L | $q_{\rm L}(t)$ | $1-\alpha_{\rm A}^{-1}p_{\rm O}(t+\theta_{\rm P})-\int_0^{t+\theta_{\rm P}} p_{\rm O}(t_1)\,dt_1$ |
| Pre-symptomatic Infectious $A_1$ | $q_{\rm A_1}(t)$ | $\alpha_{\rm A}^{-1}p_{\rm O}(t+\theta_{\rm P})$ |
| Pre-symptomatic Infectious $A_2$ | $q_{\rm A_2}(t)$ | $\int_t^{t+\theta_{\rm P}} p_{\rm O}(t_1)\,dt_1$ |
| Symptomatic S | $q_{\rm S}(t)$ | $\int_0^t p_{\rm O}(t_1)\,dt_1$ |

## 1.3 Exponential growth/decay

The self-sustained growth rate $\lambda$ of an epidemic can be obtained by seeking a solution $X(T) = e^{\lambda(T-T_0)}$ to Eq. (S7). Simple algebra gives

$$\tilde{r}(\lambda) = 1. \tag{S10}$$

In the epidemiological literature, it is customary to express the growth rate $\lambda$ in terms of the mean reproduction number

$$R_{\rm E} = \int_0^\infty r(t)dt \equiv \tilde{r}(0) = \frac{\beta_{\rm A}}{\alpha_{\rm A}} + \tilde{\beta}_{\rm B}(0), \tag{S11}$$

where we have used Eq. (S9). The first term on the right-hand-side gives the contribution to $R_{\rm E}$ from the pre-symptomatic $A_1$ phase, and the second term from the rest. Alternatively, noting that

$$\tilde{\beta}_{\rm B}(0) \equiv \int_0^\infty \beta_{\rm B}(t)dt = \int_0^{\theta_{\rm P}} \beta_{\rm B}(t)dt + \int_{\theta_{\rm P}}^\infty \beta_{\rm B}(t)dt,$$

we may write $R_{\rm E} = R_{\rm E}^{\rm A} + R_{\rm E}^{\rm B}$, with

$$R_{\rm E}^{\rm A} = \frac{\beta_{\rm A}}{\alpha_{\rm A}} + \int_0^{\theta_{\rm P}} \beta_{\rm B}(t)dt, \qquad R_{\rm E}^{\rm B} = \int_{\theta_{\rm P}}^\infty \beta_{\rm B}(t)dt$$

being the mean reproduction numbers in the pre-symptomatic and symptomatic phases, respectively.

Dividing both sides of (S10) by $R_{\rm E}$ and making use of Eqs. (S9) and (S11), we obtain upon inversion,

$$R_{\rm E} = \Big(\frac{e^{-\lambda\theta_{\rm P}}}{\tilde{p}_{\rm O}(\lambda)}\Big)\Big(\frac{\beta_{\rm A}+\alpha_{\rm A}\tilde{\beta}_{\rm B}(0)}{\beta_{\rm A}+\alpha_{\rm A}\tilde{\beta}_{\rm B}(\lambda)}\Big). \tag{S12}$$

At $\lambda = 0$, $R_{\rm E} = 1$, as required, independent of the model parameters.

Equation (S12) defines the fundamental relation between the mean reproduction number of infected individuals and the growth rate of the epidemic. The distribution $p_{\rm O}(t)$ plays an essential role in this relation. One immediate result from Eq. (S12) is that $R_{\rm E} = 0$ is at the pole of $\tilde{p}_{\rm O}(\lambda)$. For an exponentially decaying $p_{\rm O}(t) \sim e^{-\lambda t}$ at large $t$, the pole is at $\lambda = -\lambda_O$, which yields the rate of decay when transmission stops completely.

Wallinga and Lipsitch [5] proposed a general equation between $R_{\rm E}$ and $\lambda$ based on the normalised "generation interval distribution",

$$g(t) = r(t)/R_{\rm E}. \tag{S13}$$

At the observed epidemic growth rate $\lambda$, each individual produces $R_{\rm E}(\lambda)$ offspring. Consequently,

$$\tilde{g}(\lambda) = \frac{\tilde{r}(\lambda)}{R_{\rm E}} = \frac{1}{R_{\rm E}},$$

known as the Lotka–Euler estimating equation. This equation is equivalent to (S12).

**Supplementary Table 2: Percentage of the infected population in each of the disease phases when the epidemic grows at a rate $\lambda$.**

| Phase | Probability | Expression |
|---|---|---|
| Latent L | $Q_{\mathrm{L}}(\lambda)$ | $1-\left(1+\frac{\lambda}{\alpha_{\mathrm{A}}}\right)e^{\lambda\theta_{\mathrm{P}}}\tilde{p}_{\mathrm{O}}(\lambda)$ |
| Pre-symptomatic Infectious $\mathrm{A}_1$ | $Q_{\mathrm{A}_1}(\lambda)$ | $\frac{\lambda}{\alpha_{\mathrm{A}}}e^{\lambda\theta_{\mathrm{P}}}\tilde{p}_{\mathrm{O}}(\lambda)$ |
| Pre-symptomatic Infectious $\mathrm{A}_2$ | $Q_{\mathrm{A}_2}(\lambda)$ | $\left(e^{\lambda\theta_{\mathrm{P}}}-1\right)\tilde{p}_{\mathrm{O}}(\lambda)$ |
| Symptomatic S | $Q_{\mathrm{S}}(\lambda)$ | $\tilde{p}_{\mathrm{O}}(\lambda)$ |

## 1.4 Percentage of subpopulations during exponential growth

Let $J_{\mathrm{L}}(T) = J_{\mathrm{L}}(T_0)e^{\lambda(T-T_0)}$ be the flux of newly infected individuals. The population size in each phase can be expressed as Laplace transforms of expressions in Supplementary Table 1,

$$
\begin{aligned}
L(T) &= \int_0^\infty q_{\mathrm{L}}(t)J_{\mathrm{L}}(T-t)dt = \left[\frac{1}{\lambda}-\left(\frac{1}{\alpha_{\mathrm{A}}}+\frac{1}{\lambda}\right)e^{\lambda\theta_{\mathrm{P}}}\tilde{p}_{\mathrm{O}}(\lambda)\right]J_{\mathrm{L}}(T),\\
A_1(T) &= \int_0^\infty q_{\mathrm{A}_1}(t)J_{\mathrm{L}}(T-t)dt = \frac{1}{\alpha_{\mathrm{A}}}e^{\lambda\theta_{\mathrm{P}}}\tilde{p}_{\mathrm{O}}(\lambda)J_{\mathrm{L}}(T),\\
A_2(T) &= \int_0^\infty q_{\mathrm{A}_2}(t)J_{\mathrm{L}}(T-t)dt = \frac{1}{\lambda}\left(e^{\lambda\theta_{\mathrm{P}}}-1\right)\tilde{p}_{\mathrm{O}}(\lambda)J_{\mathrm{L}}(T),\\
S(T) &= \int_0^\infty q_{\mathrm{S}}(t)J_{\mathrm{L}}(T-t)dt = \frac{1}{\lambda}\tilde{p}_{\mathrm{O}}(\lambda)J_{\mathrm{L}}(T).
\end{aligned}
\tag{S14}
$$

For easy reference, the percentages of subpopulations are collected in Supplementary Table 2.

## 2 Model Calibration against Case Studies

In this section, we present technical details on the estimation of model parameters from case studies reported in the literature. In line with our modelling framework, we first examine the statistics of the symptom onset time and perform a maximum likelihood analysis to determine a parametric representation of the data. We then determine the mean infectiousness of COVID-19 patients from the serial interval statistics collected during the early days of the epidemic in China. These analyses yield estimates for the transmission parameters introduced in the previous section.

### 2.1 Symptom onset time distribution

The incubation periods of individual patients before symptom onset were summarised in three articles: 59 cases collected by Men *et al.*[6], 105 cases collected by Xia *et al.* [7], and 181 cases collected by Bi *et al.* [8]. In total, we examined $N = 347$ cases with their incubation periods. In most cases, the infection date can only be assigned to a time interval of more than one day. Therefore, the actual incubation period falls between $\mathrm{IPl}_i$ and $\mathrm{IPu}_i$, $i = 1, ..., N$, where $\mathrm{IPl}_i$ and $\mathrm{IPu}_i$ set the lower and upper bounds for the incubation period of case $i$, respectively.

The raw statistics of the three datasets is shown in Main Text Figure 2a. This is done by simply assign equal weight to the possible values within the reported window $[\mathrm{IPl}_i, \mathrm{IPu}_i]$ for each of the patients. The nominal probability distribution of the incubation period from each dataset (or the aggregated one) is then obtained by taking a simple average over all the cases within the set. Although there is general consistency with regard to the overall shape of the distributions obtained, there are also significant deviations particularly outside the peak region. While part of the variations can be attributed to statistical fluctuations when the sample size is limited, it is also plausible that systematic bias exists under the equal probability assumption when dealing with uncertainties related to finite exposure windows.

Below we perform a more refined analysis, i.e., maximum likelihood estimation, of the underlying symptom onset time distribution $p_{\mathrm{O}}(\theta, t)$, with $\theta$ being the parameter set. Following the scheme proposed by Reich *et al.* [9], we consider the following likelihood function,

$$\begin{aligned} L(\theta; \mathbf{IP}) &= \prod_{i=1}^{N} L(\theta; \mathrm{IPl}_i, \mathrm{IPu}_i), \\ L_i = L(\theta; \mathrm{IPl}_i, \mathrm{IPu}_i) &= \int_{\mathrm{IPl}_i - 0.5}^{\mathrm{IPu}_i + 0.5} p_{\mathrm{O}}(\theta, t) dt. \end{aligned} \tag{S15}$$

Eyeballing the data in Main Text Figure 2a, we hypothesise the tail of the distribution to be an exponential function. This motivates us to consider the following two hybrid functional forms for $p_{\mathrm{O}}(\theta, t)$ and perform the maximum likelihood estimation.

#### 2.1.1 Hybrid log-normal distribution with an exponential tail

The log-normal distribution is commonly used for the incubation period in the epidemiological literature. We connect it to a simple exponential decay at $t_{\mathrm{e}}$:

$$p_{\mathrm{O}}(t) = \begin{cases} A p_{\ln}(t), & t \le t_{\mathrm{e}}, \\ A p_{\ln}(t_{\mathrm{e}}) e^{-\gamma(t - t_{\mathrm{e}})}, & t \ge t_{\mathrm{e}}. \end{cases} \tag{S16}$$

Here the log-normal probability density function (PDF) $p_{\ln}(t)$ is parametrised by $\mu$ and $\sigma$:

$$p_{\ln}(t) = \frac{1}{t\sigma\sqrt{2\pi}} \exp\left[-\frac{(\ln t - \mu)^2}{2\sigma^2}\right]. \tag{S17}$$

Normalisation condition of $p_{\rm O}(t)$ yields

$$A = \frac{2}{1+\mathrm{erf}\left(\frac{\ln t_{\rm e}-\mu}{\sqrt{2}\sigma}\right)+\frac{2p_{\rm ln}(t_{\rm e})}{\gamma}} \tag{S18}$$

where erf$(x)$ is the Gauss error function. Continuity of the derivatives at $t_{\rm e}$ demands

$$\gamma = \frac{p'_{\rm ln}(t_{\rm e})}{p_{\rm ln}(t_{\rm e})}. \tag{S19}$$

Therefore, we have in total three independent parameters $\theta = (t_{\rm e}, \mu, \sigma)$ in the above model. Through numerically optimising the likelihood function with the Nelder-Mead simplex algorithm, we obtained the optimal parameter sets for different $t_{\rm e}$, which is shown in Supplementary Table 3.

**Supplementary Table 3:** Maximum likelihood estimation of parameters in Eq. (S16). Results are from bootstrap re-sampling of the data described in the text, with 1000 realisations (95% confidence interval shown in parentheses).

| $t_{\rm e}$ | $-\ln L$ | $\gamma$ (day$^{-1}$) | $\mu$ | $\sigma$ |
|---|---|---|---|---|
| 4 | 404.782 | 0.290<br>(0.264,0.320) | 1.349<br>(0.416,0.568) | 0.493<br>(1.324,1.372) |
| 5 | 404.034 | 0.300<br>(0.269,0.333) | 1.460<br>(1.424,1.496) | 0.551<br>(0.487,0.616) |
| ***6** | 403.301 | 0.308<br>(0.273,0.346) | 1.512<br>(1.462,1.559) | 0.578<br>(0.517,0.643) |
| 7 | 403.897 | 0.312<br>(0.279,0.351) | 1.535<br>(1.483,1.587) | 0.591<br>(0.532,0.647) |
| 8 | 404.344 | 0.315<br>(0.282,0.351) | 1.543<br>(1.487,1.599) | 0.596<br>(0.540,0.647) |
| $\infty$<br>Log-normal | 405.564 | N/A | 1.537<br>(1.479,1.597) | 0.580<br>(0.535,0.623) |

The maximal likelihood is achieved at $t_{\rm e} = 6$. It should be noted that the exponent $\gamma$ is quite stable around 0.31/day. The bottom row gives results for the full log-normal distribution ($t_{\rm e} = \infty$), which has a smaller likelihood value than the hybrid one at $t_{\rm e} = 6$.

### 2.1.2 Hybrid Weibull distribution with an exponential tail

For the second class of functions, we use the Weibull distribution to connect with an exponential tail:

$$p_{\rm O}(t) = \begin{cases} Ap_{\rm wb}(t), & t \le t_{\rm e}, \\ Ap_{\rm wb}(t_{\rm e})e^{-\gamma(t-t_{\rm e})}, & t \ge t_{\rm e}. \end{cases} \tag{S20}$$

Here the Weibull PDF $p_{\rm wb}(t)$ is parametrised by $k$ (shape parameter) and $\lambda$ (scale parameter):

$$p_{\rm wb}(t) = \frac{k}{\lambda}\left(\frac{t}{\lambda}\right)^{k-1} \exp\left[-\left(\frac{t}{\lambda}\right)^{k}\right]. \tag{S21}$$

The normalisation factor is

$$A = \frac{1}{1-\exp\left[-\left(\frac{t_{\rm e}}{\lambda}\right)^{k}\right]+\frac{p_{\rm wb}(t_{\rm e})}{\gamma}}. \tag{S22}$$

Continuity of derivatives at $t_\mathrm{e}$ yields

$$\gamma = \frac{p'_\mathrm{wb}(t_\mathrm{e})}{p_\mathrm{wb}(t_\mathrm{e})} \tag{S23}$$

This model has three independent parameters $\theta = (t_\mathrm{e}, k, \lambda)$. We numerically optimised the likelihood function through the Nelder-Mead simplex algorithm at different $t_\mathrm{e}$, with results given in Supplementary Table 4.

**Supplementary Table 4:** Maximum likelihood estimation of parameters in Eq. (S20). Results are from bootstrap re-sampling of the data described in the text, with 1000 realisations (95% confidence interval shown in parentheses).

| $t_\mathrm{e}$ | $-\ln L$ | $\gamma$ (day$^{-1}$) | $k$ | $\lambda$ (day) |
|---|---|---|---|---|
| 3 | 405.991 | 0.283 (0.258,0.311) | 4.137 (3.409,5.029) | 3.029 (0.258,0.311) |
| ***4** | 404.332 | 0.303 (0.274,0.335) | 3.118 (2.701,3.619) | 3.917 (3.872,3.958) |
| 5 | 405.557 | 0.320 (0.287,0.357) | 2.638 (2.355,2.968) | 4.627 (4.542,4.711) |
| 6 | 406.088 | 0.338 (0.302,0.379) | 2.363 (2.146,2.608) | 5.150 (5.014,5.286) |
| 7 | 406.878 | 0.357 (0.317,0.403) | 2.189 (2.010,2.386) | 5.518 (5.337,5.704) |
| $\infty$ Weibull | 410.949 | N/A | 1.828 (1.707,1.958) | 6.206 (5.885,6.554) |

The maximal likelihood is achieved at $t_\mathrm{e} = 4$. The likelihood for $t_\mathrm{e} = \infty$ is lower which suggests that our hybrid function is more faithful to the data than the full Weibull distribution.

The estimated value for $\gamma$ here is very similar to the result presented in Supplementary Table 3. However, the optimal likelihood obtained under the hybrid Weibull distribution is quite a bit less than that of the hybrid log-normal distribution. Therefore, we adopt the log-normal distribution with an exponential tail as the best estimate for $p_\mathrm{O}(t)$ and use it in our numerical calculations.

#### 2.1.3 Uncertainty analysis through bootstrap re-sampling

Confidence intervals given in Supplementary Tables 3 and 4 are obtained following a bootstrap scheme[9]. This is done by generating 1000 re-sampled copies of the initial 347 cases dataset. The maximum likelihood estimation is then performed for each of the re-sampled copy. Results for $p_\mathrm{O}(t)$ so estimated are shown as the grey lines in Main Text Figure 2a.

### 2.2 Transmission characteristics

#### 2.2.1 Infectiousness around symptom onset

Records of disease transmission by individual patients through the course of their disease progression are scarce in the public domain. We therefore estimated the transmission parameters defined in Supplementary Figure 1 indirectly using the reported infector-infectee pairs. Such a procedure is subject to bias arising from the data collection process. For example, intra-family transmission tends to be over-represented[10, 11]. Keeping potential shortcomings of this type in mind, we present below quantification of our model using the data provided by He *et al.* [12]

The dataset contains 74 infector-infectee pairs. Among them, 66 pairs have a unique symptom onset date (see Source Data). They are used in our analysis. As illustrated in Supplementary Figure 2, each transmission pair $i$ is associated with an exposure window $\mathrm{W}_i = [\mathrm{Wl}_i, \mathrm{Wu}_i]$, where $\mathrm{Wl}_i$ and $\mathrm{Wu}_i$ are integers that together specify an exposure window around the symptom onset of the infector (see Supplementary Figure 2).

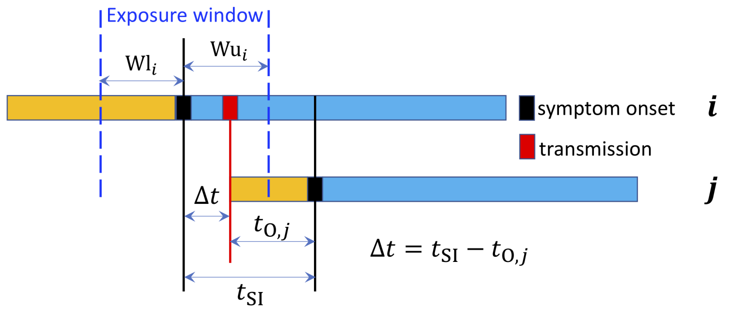


**Supplementary Figure 2: Temporal events in a pairwise disease transmission.** The index patient $i$ transmits the virus to infectee $j$ within an exposure window measured from the symptom onset of patient $i$. The two symptom onsets are indicated by black bars along the time axis, while the actual transmission event is indicated by the red bar. $t_{\mathrm{SI}}$ is the serial interval, $t_{\mathrm{O},j}$ is the incubation period of infectee $j$. $\Delta t$ is the time difference between the transmission event and the symptom onset of infector $i$, which can be either negative (pre-symptomatic transmission) or positive (post-symptomatic transmission). $\mathrm{Wl}_i$ and $\mathrm{Wu}_i$ are the left and right bounds of the exposure window, w.r.t the symptom onset of infector $i$.

Below, we quantify the statistics of disease transmission time with a probability density function $p_{\mathrm{I}}(\theta, t)$, with $t$ measured from the symptom onset of the infector, and $\theta$ the set of parameters to be determined. Then maximum likelihood estimation is applied to fit the discrete exposure window data [9]. The likelihood function is given by:

$$
\begin{aligned}
L(\theta; \mathbf{W}) &= \prod_{i=1}^{N} L(\theta; \mathrm{W}_i), \\
L_i = L(\theta; \mathrm{W}_i) &= \int_{\mathrm{Wl}_i - 0.5}^{\mathrm{Wu}_i + 0.5} p_{\mathrm{I}}(\theta, t) dt.
\end{aligned}
\tag{S24}
$$

Given the limited resolution for the exposure window in integral values, we shall limit ourselves to the following parametrisation of $p_{\mathrm{I}}(\theta, t)$: a conglomerate of two exponential wings and its extensions with either a flat or a smooth cap. We consider each of them separately.

### 2.2.2 A conglomerate of two exponential wings

We first consider the case of two exponential functions joined directly at $\theta_{\mathrm{P}}$:

$$
p_{\mathrm{I}}(\theta, t) = \begin{cases} A e^{\alpha_{\mathrm{A}}(t - \theta_{\mathrm{P}})}, & t \le \theta_{\mathrm{P}}, \\ A e^{-\alpha_{\mathrm{B}}(t - \theta_{\mathrm{P}})}, & t \ge \theta_{\mathrm{P}}. \end{cases}
\tag{S25}
$$

This model has three independent parameters $\theta = (\alpha_{\mathrm{A}}, \alpha_{\mathrm{B}}, \theta_{\mathrm{P}})$. The normalisation factor $A$ is simply

$$
A = \frac{\alpha_{\mathrm{A}} \alpha_{\mathrm{B}}}{\alpha_{\mathrm{A}} + \alpha_{\mathrm{B}}}.
\tag{S26}
$$

By numerically optimising the likelihood function through the Nelder-Mead simplex algorithm, we obtained the optimal parameter set shown in Supplementary Table 5.

**Supplementary Table 5:** Maximum likelihood estimation of the parameters in Eq. (S25) with 1000 bootstrap re-samplings (95% confidence interval shown in parentheses).

| $-\ln L$ | $\alpha_{\rm A}$ | $\alpha_{\rm B}$ | $\theta_{\rm P}$ |
|---|---|---|---|
| 55.551 | 0.434 (0.320,0.692) | 0.541 (0.475,0.652) | −0.677 (−1.019,−0.124) |

#### 2.2.3 Exponential wings with a flat cap

Expanding the peak of the function defined by (S25) into a flat cap of size $\epsilon$, we have,

$$p_{\rm I}(\theta,t)=\begin{cases}Ae^{\alpha_{\rm A}(t-\theta_{\rm P1})}, & t\le\theta_{\rm P1},\\ A, & \theta_{\rm P1}\le t\le\theta_{\rm P2},\\ Ae^{-\alpha_{\rm B}(t-\theta_{\rm P2})}, & t\ge\theta_{\rm P2}.\end{cases} \tag{S27}$$

Here,

$$\begin{aligned}\theta_{\rm P1}&=\theta_{\rm P}-\frac{\epsilon}{2},\\ \theta_{\rm P2}&=\theta_{\rm P}+\frac{\epsilon}{2}.\end{aligned} \tag{S28}$$

The normalisation factor is simply

$$A=\frac{1}{\frac{1}{\alpha_{\rm A}}+\frac{1}{\alpha_{\rm B}}+\epsilon}. \tag{S29}$$

This model has four independent parameters $\theta=(\alpha_{\rm A},\alpha_{\rm B},\theta_{\rm P},\epsilon)$.

Through numerical optimisation of the likelihood functions at different values of $\epsilon$, we obtained the optimal parameter sets, which are shown in Supplementary Table 6.

**Supplementary Table 6:** Maximum likelihood estimation of parameters in Eq. (S27).

| $\epsilon$ | $-\ln L$ | $\alpha_{\rm A}$ | $\alpha_{\rm B}$ | $\theta_{\rm P}$ | $\theta_{\rm P1}$ | $\theta_{\rm P2}$ |
|---|---|---|---|---|---|---|
| 2.0 | 56.705 | 0.417 | 0.596 | −0.460 | −1.460 | 0.540 |
| 1.0 | 55.907 | 0.428 | 0.558 | −0.630 | −1.130 | −0.130 |
| 0.5 | 55.660 | 0.434 | 0.543 | −0.697 | −0.947 | −0.447 |
| 0.2 | 55.569 | 0.434 | 0.541 | −0.690 | −0.790 | −0.590 |
| 0.1 | 55.555 | 0.434 | 0.541 | −0.683 | −0.733 | −0.633 |
| 0 | 55.551 | 0.434 | 0.541 | −0.677 | −0.677 | −0.677 |

It can be seen from the above table that the likelihood increases with decreasing cap width $\epsilon$, reaching its maximum at $\epsilon=0$ which is treated above.

#### 2.2.4 Exponential wings with a smooth cap

We now consider the case of a smooth cap as defined by:

$$p_{\rm I}(\theta,t)=\begin{cases}A\left[1-\chi\left(\theta_{\rm P1}-\theta_{\rm P}\right)^2\right]e^{\alpha_{\rm A}(t-\theta_{\rm P1})}, & t\le\theta_{\rm P1},\\ A\left[1-\chi\left(t-\theta_{\rm P}\right)^2\right], & \theta_{\rm P1}\le t\le\theta_{\rm P2},\\ A\left[1-\chi\left(\theta_{\rm P2}-\theta_{\rm P}\right)^2\right]e^{-\alpha_{\rm B}(t-\theta_{\rm P2})}, & t\ge\theta_{\rm P2}.\end{cases} \tag{S30}$$

The shape of the quadratic function in the middle is parametrised by $\chi$, with its peak located at $\theta_{\mathrm{P}}$. The normalisation factor is

$$A = \frac{1}{(\theta_{\mathrm{P2}} - \theta_{\mathrm{P1}}) - \frac{\chi}{3}\left[(\theta_{\mathrm{P2}} - \theta_{\mathrm{P}})^3 + (\theta_{\mathrm{P}} - \theta_{\mathrm{P1}})^3\right] + \frac{1}{\alpha_{\mathrm{A}}}\left[1 - \chi(\theta_{\mathrm{P1}} - \theta_{\mathrm{P}})^2\right] + \frac{1}{\alpha_{\mathrm{B}}}\left[1 - \chi(\theta_{\mathrm{P2}} - \theta_{\mathrm{P}})^2\right]}. \tag{S31}$$

Smoothness requires continuity of derivatives at $\theta_{\mathrm{P1}}$ and $\theta_{\mathrm{P2}}$, respectively, which yields:

$$\begin{aligned} \alpha_{\mathrm{A}} &= \frac{2\chi(\theta_{\mathrm{P}} - \theta_{\mathrm{P1}})}{1 - \chi(\theta_{\mathrm{P}} - \theta_{\mathrm{P1}})^2}, \quad \theta_{\mathrm{P1}} = \theta_{\mathrm{P}} + \frac{1}{\alpha_{\mathrm{A}}} - \sqrt{\frac{1}{\alpha_{\mathrm{A}}^2} + \frac{1}{\chi}}, \\ \alpha_{\mathrm{B}} &= \frac{2\chi(\theta_{\mathrm{P2}} - \theta_{\mathrm{P}})}{1 - \chi(\theta_{\mathrm{P2}} - \theta_{\mathrm{P}})^2}, \quad \theta_{\mathrm{P2}} = \theta_{\mathrm{P}} - \frac{1}{\alpha_{\mathrm{B}}} + \sqrt{\frac{1}{\alpha_{\mathrm{B}}^2} + \frac{1}{\chi}}. \end{aligned} \tag{S32}$$

Therefore, there are four independent parameters $\theta = (\alpha_{\mathrm{A}}, \alpha_{\mathrm{B}}, \theta_{\mathrm{P}}, \chi)$. Note that in the limit of $\chi \to \infty$ this model reduces to the first model with two exponential functions joining at $\theta_{\mathrm{P}}$.

We numerically optimised the likelihood functions through the Nelder-Mead simplex algorithm at different $\chi$. Optimal parameter sets in each case are shown in Supplementary Table 7.

**Supplementary Table 7:** Maximum likelihood estimation of parameters in Eq. (S30).

| $\chi$ | $-\ln L$ | $\alpha_{\mathrm{A}}$ | $\alpha_{\mathrm{B}}$ | $\theta_{\mathrm{P}}$ | $\theta_{\mathrm{P1}}$ | $\theta_{\mathrm{P2}}$ |
|---|---|---|---|---|---|---|
| 0.2 | 56.018 | 0.419 | 0.551 | −0.672 | −1.556 | 0.392 |
| 0.5 | 55.662 | 0.431 | 0.541 | −0.707 | −1.104 | −0.228 |
| 1.0 | 55.584 | 0.434 | 0.540 | −0.704 | −0.912 | −0.452 |
| 5.0 | 55.552 | 0.434 | 0.541 | −0.683 | −0.726 | −0.629 |
| 10.0 | 55.551 | 0.434 | 0.541 | −0.680 | −0.702 | −0.653 |
| $\infty$ | 55.551 | 0.434 | 0.541 | −0.677 | −0.677 | −0.677 |

Note that the likelihood increases with increasing $\chi$, which indicates that the optimal estimation is achieved at vanishing cap size.

In Main Text Figure 2b, we show the infectiousness curves obtained from the first model, the second model at $\epsilon = 1.0$, and the third model at $\chi = 1.0$.

### 2.2.5 Uncertainty analysis through bootstrap re-sampling

We performed bootstrap analysis to determine uncertainties in the estimated $p_{\mathrm{I}}(t)$. This is done by generating 1000 re-sampled copies of the exposure window dataset of 66 transmission pairs. The maximum likelihood estimation for $p_{\mathrm{I}}(t)$ is then performed for each of the re-sampled copy. The $p_{\mathrm{I}}(t)$ for each of the re-sampled copied are shown as the grey lines in Main Text Figure 2b (for the conglomerate of two exponentials). The 95% confidence intervals for the estimated parameters are shown in Supplementary Table 5 and the Main Text Table I.

## 2.3 Serial interval statistics

Du *et al.* [10], Xu *et al.* [11], and He *et al.* [12] reported the statistics of the serial intervals of infector-infectee pairs in 468, 679, and 66 confirmed serial infection cases, respectively, which we use to compare against the symptom onset distribution function as well as the infectiousness function obtained above.

In a pairwise transmission event $i \to j$, the serial interval $t_{\rm SI}$ is the sum of the time of transmission $\Delta t$ with respect to the symptom onset of the index patient $i$, and the symptom onset time $t_{{\rm O},j}$ of the infectee $j$, as illustrated in Supplementary Figure 2. Assuming statistical independence between the latter two in the clinical samples collected, we may write the serial interval distribution as a convolutional integral,

$$p_{\rm SI}(t_{\rm SI}) = \int_{-\infty}^{t_{\rm SI}} p_{\rm I}(\Delta t) p_{\rm O}(t_{\rm SI} - \Delta t) d\Delta t. \quad \text{(S33)}$$

In Main Text Figure 2c, we show the calculated serial interval distribution (red line) using Eq. (S33) and the estimated $p_{\rm O}(t)$ [Eq. (S16) at $t_{\rm e} = 6$] and $p_{\rm I}(\Delta t)$ [Eq. (S25), with $t$ replaced by $\Delta t$], with the parameter values given in Supplementary Tables 3 and 5 and Table I in the Main Text. As noted in earlier studies [11, 13], the empirical serial interval distributions can be skewed by shifts in the transmission window of the index patients surveyed. For example, travel delays move the effective $p_{\rm I}(\Delta t)$ in the positive direction, while isolation of symptomatic patients move the effective $p_{\rm I}(\Delta t)$ in the negative direction. In each case, $p_{\rm SI}(t_{\rm SI})$ shifts correspondingly in the same direction.

## 2.4 Non-Markovian transition rate from latent to infectious phase

With the symptom onset time distribution $p_{\rm O}(t)$ and the parameters $\alpha_{\rm A}$ and $\theta_{\rm P}$ estimated from clinical and transmission case studies, we may proceed to compute the probabilities for an infected person to be in each of the disease phases on day $t$ since infection. This can be done by numerically evaluating the expressions presented in Supplementary Table 1. Supplementary Figure 3 shows the probability $q_{\rm L}(t)$ for the latent phase and the corresponding exit rate $\alpha_{\rm L}(t)$. Good agreement is seen for $q_{\rm L}(t)$ under the two parametrisation schemes adopted. The curves for $\alpha_L(t) = -\dot{q}_L(t)/q_{\rm L}(t)$ also exhibit the same trend, although discrepancies exist in the actual shape. On the first day since infection, the transition rate to the infectious phase is low which explains the low value of $\alpha_{\rm L}(t)$ in this period. This is followed by an accelerated transition rate and a faster than exponential decay of $q_{\rm L}(t)$, reaching a peak value of 0.6 day$^{-1}$ or above. After that the transition rate decelerates and reaches a plateau value of approximately 0.3 day$^{-1}$ on the fifth day onward. Given that both $\alpha_{\rm A}$ and $\alpha_{\rm B}$ as given in Supplementary Table 5 are greater than 0.4 day$^{-1}$, we see that the long-time tails of the incubation period $p_{\rm O}(t)$ and the serial interval distribution $p_{\rm SI}(t)$ (Main Text Figure 2c) can be attributed to the long-time tail of the latent phase.

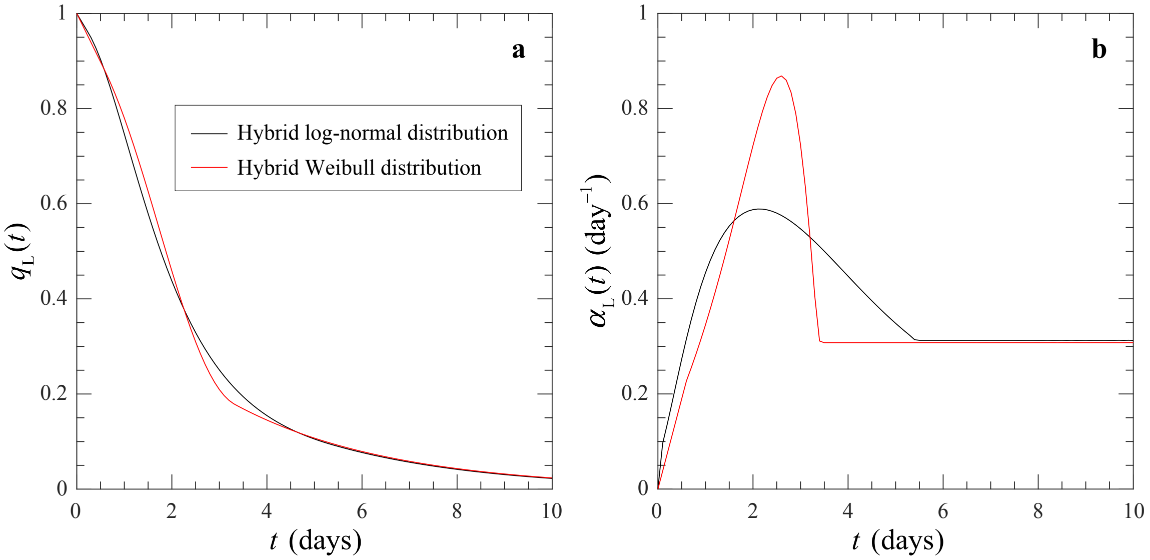


**Supplementary Figure 3: Non-Markovian exit rates from the latent phase.** a. Probability of an individual to remain in the latent phase on day $t$ since infection. b. Rate to exit from the latent phase against $t$. The curves are computed numerically from the equation for $q_{\rm L}(t)$ given in Supplementary Table 1, using the estimated incubation period distribution $p_{\rm O}(t)$ of this section (black: hybrid log-normal distribution; red: hybrid Weibull distribution). Values for $\alpha_{\rm A}$ and $\theta_{\rm P}$ are given in Supplementary Table 5.

# 3 Simplifying Approximations

In Sec. 1 we expressed various quantities in terms of the Laplace transform of $p_{\rm O}(t)$. Here we consider simplifying approximations, which allow for more direct relations to be derived.

## 3.1 An approximate formula for the mean reproduction rate

As an explicit example, we consider disease transmission in a homogeneous population following the infectiousness curve shown in Main Text Figure 2b. The peak infectiousness happens at $\theta_{\rm P} = -0.68$ days before the symptom onset, yielding $\theta_{\rm P} = 0.68$ days. The left and right wings are well fitted to exponential functions with decay rates $\alpha_{\rm A} \simeq 0.43$ day$^{-1}$ and $\alpha_{\rm B} \simeq 0.54$ day$^{-1}$, respectively. For the right wing, we may write

$$\beta_{\rm B}(t_{\rm B}) = \beta_{\rm A} \exp(-\alpha_{\rm B} t_{\rm B}), \tag{S34}$$

where $t_{\rm B}$ is measured from the beginning of the $A_2$ phase, e.g., $\theta_{\rm P}$ days before the symptom onset. The mean infectiousness curve on the left also matches well with the exponential distribution of the duration of the $A_1$ phase in the model introduced in Sec. 1, with a decay rate $\alpha_{\rm A}$.

From Eq. (S34), we obtain the Laplace transform,

$$\tilde{\beta}_{\rm B}(\lambda) = \int_0^\infty \beta_{\rm A} e^{-\alpha_{\rm B} t} e^{-\lambda t} dt = \frac{\beta_{\rm A}}{\lambda + \alpha_{\rm B}}. \tag{S35}$$

Substituting Eq. (S35) into (S9), we obtain,

$$\tilde{r}(\lambda) = \left(\frac{\beta_{\rm A}}{\alpha_{\rm A}} + \frac{\beta_{\rm A}}{\lambda + \alpha_{\rm B}}\right) e^{\lambda \theta_{\rm P}} \tilde{p}_{\rm O}(\lambda). \tag{S36}$$

For $\lambda \ll \alpha_{\rm B}$, we may approximate

$$1+\frac{\alpha_{\rm A}}{\lambda+\alpha_{\rm B}} \simeq \left(1+\frac{\alpha_{\rm A}}{\alpha_{\rm B}}\right)\left(1-\frac{\alpha_{\rm A}}{\alpha_{\rm B}}\frac{\lambda}{\alpha_{\rm A}+\alpha_{\rm B}}\right) \simeq \left(1+\frac{\alpha_{\rm A}}{\alpha_{\rm B}}\right) e^{-\frac{\lambda\alpha_{\rm A}}{\alpha_{\rm B}(\alpha_{\rm A}+\alpha_{\rm B})}}.$$

Introducing

$$\theta_{\rm S} = \theta_{\rm P} - \frac{\alpha_{\rm A}}{\alpha_{\rm B}(\alpha_{\rm A}+\alpha_{\rm B})}, \tag{S37}$$

we may then write,

$$\tilde{r}(\lambda) \simeq \left(\frac{\beta_{\rm A}}{\alpha_{\rm A}}+\frac{\beta_{\rm A}}{\alpha_{\rm B}}\right) e^{\lambda\theta_{\rm S}}\tilde{p}_{\rm O}(\lambda). \tag{S38}$$

This yields a very simple expression for the reproduction rate,

$$r(t) \simeq R_{\rm E} p_{\rm O}(t+\theta_{\rm S}), \tag{S39}$$

with $R_{\rm E} = (\beta_{\rm A}/\alpha_{\rm A}) + (\beta_{\rm A}/\alpha_{\rm B})$. Equation (S13) then identifies $p_{\rm O}(t+\theta_{\rm S})$ with the generation time interval distribution $g(t)$.

A few remarks with regard to the re-parameterised model (S39) are in order. The parameter $R_{\rm E}$, which sets the overall scale of transmission, incorporates total contribution from both pre-symptomatic and symptomatic individuals (Main Text Figure 1). The shift parameter $\theta_{\rm S}$, on the other hand, depends on how fast transmission decays on the symptomatic side. As seen from Eq. (S37), a small decay rate $\alpha_{\rm B}$ may change $\theta_{\rm S}$ into the negative. Using the parameter values determined from Sec. 2, we obtain $\theta_{\rm S} \simeq -0.15$ day. In this case, the peak-shift of the infectiousness curve to the pre-symptomatic side is compensated by transmission from symptomatic patients. As reported in Ref. [13], the latter could vary over time depending on the isolation measures applied to symptomatic patients.

### 3.2 A Markov model

A number of modelling studies in the literature adopt a Markovian setup with $\alpha_{\rm L}(t) = const.$ which is a special case of our more general non-Markovian approach (see, e.g., Ref.[4]). Skipping the $A_2$ phase by setting $\theta_{\rm P} = 0$, we have,

$$\tilde{q}_{\rm L} = \frac{1}{\alpha_{\rm L}+\lambda}, \quad \tilde{p}_{\rm O} = \frac{\alpha_{\rm L}}{\alpha_{\rm L}+\lambda}\frac{\alpha_{\rm A}}{\alpha_{\rm A}+\lambda}. \tag{S40}$$

The onset time distribution in this case is given by

$$p_{\rm O}(t) = \frac{\alpha_{\rm L}\alpha_{\rm A}}{\alpha_{\rm A}-\alpha_{\rm L}}\left(e^{-\alpha_{\rm L}t}-e^{-\alpha_{\rm A}t}\right) = \frac{1}{\tau_{\rm L}-\tau_{\rm A}}\left(e^{-t/\tau_{\rm L}}-e^{-t/\tau_{\rm A}}\right), \tag{S41}$$

with its mean and variance given by,

$$\tau_{\rm O} = \langle t_{\rm O}\rangle = \tau_{\rm L}+\tau_{\rm A}, \quad \sigma_{\rm O}^2 = \langle t_{\rm O}^2\rangle - \langle t_{\rm O}\rangle^2 = \tau_{\rm L}^2+\tau_{\rm A}^2,$$

and

$$\frac{\sigma_{\rm O}^2}{\tau_{\rm O}^2} = \varepsilon^2 + (1-\varepsilon)^2,$$

where $\tau_{\rm L} = \varepsilon\tau_{\rm O}$, $\tau_{\rm A} = (1-\varepsilon)\tau_{\rm O}$. In the limit $\tau_{\rm L} = \tau_{\rm A} =$ or $\varepsilon = 1/2$, $p_{\rm O}(t) = \frac{t}{\tau_{\rm L}^2}e^{-t/\tau_{\rm L}}$.

Substituting Eq. (S40) into Eq. (S9), we obtain the Laplace transform of the kernel function,

$$\tilde{r}_{\rm M}(\lambda) = \frac{\alpha_{\rm L}}{\alpha_{\rm L}+\lambda}\frac{\beta_{\rm A}+\alpha_{\rm A}\tilde{\beta}_{\rm B}(\lambda)}{\alpha_{\rm A}+\lambda}. \tag{S42}$$

Below we consider a situation where $\beta_{\rm B}(t_{\rm B})$ decays to zero rapidly, i.e., the symptomatic transmission is much reduced. The second factor on the right-hand side of Eq. (S42) can then be approximated as,

$$\frac{\beta_{\rm A}+\alpha_{\rm A}\tilde{\beta}_{\rm B}(\lambda)}{\alpha_{\rm A}+\lambda}\simeq\frac{R_{\rm E}}{1+\lambda/\alpha_{\rm A,eff}},$$

where $R_{\rm E}$ is given by Eq. (S11) and $\alpha_{\rm A,eff}=\alpha_{\rm A}[1+\alpha_{\rm A}\tilde{\beta}'_{\rm B}(0)/R_{\rm E}]$, with $\tilde{\beta}'_{\rm B}(0)$ being the derivative of $\tilde{\beta}_{\rm B}(\lambda)$ at $\lambda=0$. It follows that

$$\tilde{r}_{\rm M}(\lambda)\simeq\frac{R_{\rm E}}{\left(1+\frac{\lambda}{\alpha_{\rm L}}\right)\left(1+\frac{\lambda}{\alpha_{\rm A,eff}}\right)},\tag{S43}$$

The inverse transform of the above expression yields

$$r_{\rm M}(t)\simeq R_{\rm E}\frac{\alpha_{\rm L}\alpha_{\rm A,eff}}{\alpha_{\rm A,eff}-\alpha_{\rm L}}\left(e^{-\alpha_{\rm L}t}-e^{-\alpha_{\rm A,eff}t}\right).\tag{S44}$$

Under the effective kernel function (S43), Eq. (S10) takes the form,

$$R_{\rm E}=\left(1+\frac{\lambda}{\alpha_{\rm L}}\right)\left(1+\frac{\lambda}{\alpha_{\rm A,eff}}\right),\tag{S45}$$

i.e., a parabola with two nodes at $\lambda_L\simeq-\alpha_{\rm L}$ and $\lambda_{\rm A}\simeq-\alpha_{\rm A,eff}$.

# 4 Model Exploration under Intervention

In this section, we consider the effects of various intervention and containment measures aimed at a significant reduction of the mean reproduction number $R_{\mathrm{E}}$ from its nominal number of 3 or above in an unprotected population, within the framework of the model described in Sec. 1. When these measures are implemented in combination, reductions multiply. Some of these measures, such as social distancing and wearing face masks, are aiming at an overall reduction of COVID-19 transmission in the population, while others are targeting more specifically at breaking down the transmission chain. Considering the difficulty in achieving a three-fold reduction of $R_{\mathrm{E}}$ under any single measure, one is left with no option other than adopting as many of these measures as circumstances allow.

## 4.1 Quarantine of infected individuals

Quarantine of infected individuals cuts down disease transmission. Naturally, the biggest challenge is to identify such individuals and isolate them sufficiently to stop transmission. We consider several scenarios below.

### 4.1.1 Mean reproduction number from day $t$ onward since infection

Let $t$ be the time interval between infection and identification of a given infected individual. Supplementary Table 8 gives the mean reproduction number from day $t$ onward. The result depends on which phase the patient is in.

**Supplementary Table 8: Mean reproduction number from day $t$ onward since infection.**

| Disease Phase | Mean reproduction number |
|---|---|
| L & $A_1$ | $R_{\mathrm{E}}$ |
| entering $A_2$ at $t_1 < t$ | $\Delta R_{\mathrm{B}}(t-t_1) = \int_{t-t_1}^{\infty} \beta_{\mathrm{B}}(t_2) dt_2$ |

### 4.1.2 Testing and quarantine

Testing and quarantining of infected individuals is practiced in South Korea during the early stages of the pandemic with great intensity. In the simplest scenario, a suspected individual undergoes one time test of COVID-19 infection. It takes a day or so for the test result to come back. If it is positive, the person will be quarantined and hence removed from the active infected population. Oral nucleic acid test only reports cases with a sufficiently high viral load. Therefore the test needs to be done around the time of symptom onset. However, by then the person may have already infected other people. Within our probabilistic framework, the efficiency of this procedure can be assessed as follows.

We assume that the test protocol is implemented in such a way that a person on day $t$ since infection is tested at a rate $\eta_{\mathrm{testing}}(t)$, i.e., the probability that he/she is tested during the time interval $(t, t+dt)$ is $\eta_{\mathrm{testing}}(t)dt$, with $\int_0^{\infty} \eta_{\mathrm{testing}}(t)dt \leq 1$. We shall also assume that the test result, which is returned after $\tau_{\mathrm{d}}$ days, will be positive only when the infected has already passed the latent phase on the day of testing. With the help of the results in Supplementary Table 8, we may write the reduction in the mean reproduction number $R_{\mathrm{E}}$ at the population

level,

$$\Delta R_{\rm testing} = \int_0^\infty dt \eta_{\rm testing}(t)\Big(q_{\rm A_1}(t)\big[e^{-\alpha_{\rm A}\tau_{\rm d}}R_{\rm E} + \phi(\tau_{\rm d})\big] + \int_0^t dt_1 \alpha_{\rm A} q_{\rm A_1}(t_1)\Delta R_{\rm B}(t+\tau_{\rm d}-t_1)\Big). \tag{S46}$$

Here

$$\phi(\tau) = \int_0^\tau dt \alpha_{\rm A} e^{-\alpha_{\rm A}t}\Delta R_{\rm B}(\tau - t) = \big(1-e^{-\alpha_{\rm A}\tau}\big)\tilde{\beta}_B(0) - \int_0^\tau dt\big(1-e^{-\alpha_{\rm A}t}\big)\beta_{\rm B}(t).$$

In the extreme case that all infected are tested on day $t_{\rm test}$ of their infection, we have $\eta_{\rm testing}(t) = \delta(t - t_{\rm test})$. Under Eqs. (S34) and (S46), the relative reduction of $R_{\rm E}$ is given by,

$$\begin{aligned}\frac{\Delta R_{\rm testing}(t_{\rm test})}{R_{\rm E}} =& q_{\rm A_1}(t_{\rm test})\left(\frac{\alpha_{\rm B}}{\alpha_{\rm A}+\alpha_{\rm B}}e^{-\alpha_{\rm A}\tau_{\rm d}} + \frac{\alpha_{\rm A}}{\alpha_{\rm A}+\alpha_{\rm B}}e^{-\alpha_{\rm B}\tau_{\rm d}} + \frac{\alpha_{\rm A}\alpha_{\rm B}}{(\alpha_{\rm A}+\alpha_{\rm B})^2}\big(1-e^{-(\alpha_{\rm A}+\alpha_{\rm B})\tau_{\rm d}}\big)\right)\\ &+ \frac{\alpha_{\rm A}^2}{\alpha_{\rm A}+\alpha_{\rm B}}e^{-\alpha_{\rm B}\tau_{\rm d}}\int_0^{t_{\rm test}} q_{\rm A_1}(t_{\rm test}-t)e^{-\alpha_{\rm B}t}dt.\end{aligned} \tag{S47}$$

According to the expression in Supplementary Table 1, $q_{\rm A_1}(t_{\rm test})$ is peaked one day before the peak of the symptom onset time distribution. Consequently, the maximum reduction is also achieved when $t_{\rm test}$ is chosen to be around that day. For smaller $t_{\rm test}$, the chance of returning a positive test result is low, while for larger $t_{\rm test}$, the patient in question is likely to have already infected others by the time of the test.

#### 4.1.3 Contact tracing

In contact tracing, a high percentage of close contacts of a newly confirmed viral carrier (primary case) are identified and quarantined soon after the contact took place, without testing. This procedure is more effective when performed sufficiently close to the contact date, as it also covers secondary cases that are still in the latent phase of their disease progression. For the secondary cases, their $r(t)$ is truncated on the day they are located, while transmission to tertiary cases could happen before that time. At an overall success rate $q_{\rm c}$, we assume infectees are traced down within a time window $t_{\rm trace}$ since infection. The reduction of $R_E$ is then given by,

$$\Delta R_{\rm E} = q_{\rm c}\int_0^{t_{\rm trace}}\frac{dt}{t_{\rm trace}}\int_t^\infty r(t_1)dt_1 = q_{\rm c}R_{\rm E}\Big[1 - \frac{1}{t_{\rm trace}}\int_0^{t_{\rm trace}+\theta_{\rm S}} q_{\rm S}(t)dt\Big], \tag{S48}$$

where we have used Eq. (S39). Expression for $q_{\rm S}(t)$ is given in Supplementary Table 1.

### 4.2 Mask wearing

Considering the dual-effects of mask-wearing in reducing both virus inhalation by susceptible individuals and exhalation by infectious individuals (including pre-symptomatic and asymptomatic groups), we calculated the reduction of transmission rate $\beta$ against mask efficacy and the percentage of the population wearing masks under a simplifying approximation.

We assumed that there are COVID-19 positive (P) and negative (N) individuals. $p_{\rm m}$ is the percentage of the population that wear masks: $p_{\rm mP}$ for positive individuals and $p_{\rm mN}$ for negative individuals. Here, $e$ denotes the efficacy of masks measured by the percentage of virus trapped by the mask: from inhalation ($e_{\rm in}$ , important for COVID-19 negative individuals) and exhalation ($e_{\rm ex}$, important for COVID-19 positive individuals). In the absence of masks, the rate of transmission by a positive individual contacting a negative individual is $\beta$.

Therefore, the averaged chance of transmission is:

$$\beta_{\rm mask} = \beta(1 - e_{\rm in}\cdot p_{\rm mN})\cdot(1 - e_{\rm ex}\cdot p_{\rm mP}).$$

**Supplementary Table 9: Four types of encounters between a positive and a negative individual and reduction of the transmission rate $\beta$.**

| Prob. of contact type | P wearing mask | N wearing mask | Chance of transmission |
|---|---|---|---|
| $p_{\mathrm{mP}} \cdot p_{\mathrm{mN}}$ | Yes | Yes | $\beta(1-e_{\mathrm{ex}})(1-e_{\mathrm{in}})$ |
| $p_{\mathrm{mP}}(1-p_{\mathrm{mN}})$ | Yes | No | $\beta(1-e_{\mathrm{ex}})$ |
| $(1-p_{\mathrm{mP}})p_{\mathrm{mN}}$ | No | Yes | $\beta(1-e_{\mathrm{in}})$ |
| $(1-p_{\mathrm{mP}})(1-p_{\mathrm{mN}})$ | No | No | $\beta$ |

Under the totally symmetric assumption $e_{\mathrm{ex}} = e_{\mathrm{in}} = e$, $p_{\mathrm{mN}} = p_{\mathrm{mP}} = p_{\mathrm{m}}$, the result becomes:

$$\beta_{\mathrm{mask}} = \beta(1 - e \cdot p_{\mathrm{m}})^2. \qquad \text{(S49)}$$

Note that this equation can be applied separately to pre-symptomatic and symptomatic transmission, with their own $p_{\mathrm{m}}$. In the numerical examples presented below and in the Main Text, we used the same $p_{\mathrm{m}}$ for both types of transmission. In the more general case, the reduction of $\beta$ can be calculated when contributions of the two types to the total are known.

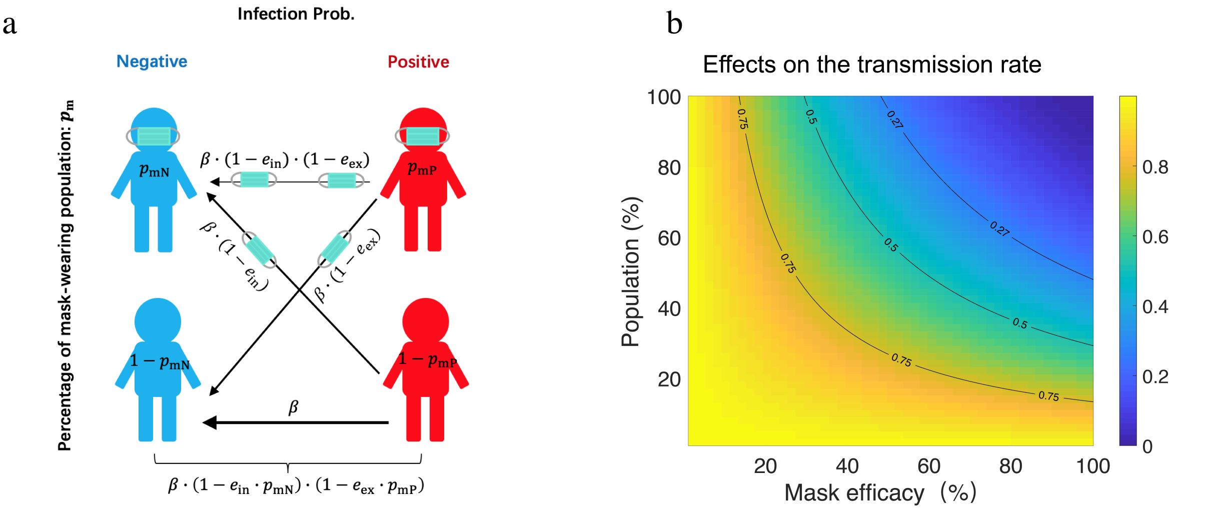


**Supplementary Figure 4: Estimation on the impact of mask-wearing on the transmission rate.** a. Schematic representation on the dual-effects of masks in reducing $\beta$. b. Relationship between the factor multiplying $\beta$ (heatmap color) with the mask efficacy ($x$-axis) and the fraction of the population wearing masks ($y$-axis). Black lines show the contours for reducing $\beta$ to 0.75, 0.5, 0.27 of its original value.

The above discussion shows, in a semi-quantitative way, that mask-wearing brings benefits to one-self if not infected but more importantly to others. Even with masks at a moderate efficacy of 50%, reduction of the transmission rate can be substantial when practiced by the whole population.

Previous research on influenza suggested that surgical mask reduces 70% of the viral aerosol shedding [14]. Also, WHO suggested that respiratory droplets ($> 5 \sim 10$ $\mu$m in diameter) and contacts are the primary routes for COVID-19 to transmit between people [15]. Surgical mask reduces more than 90% of droplets in this size range [16]. However, general public may not be able to fully comply with the usage guidance of surgical masks. Therefore, in generating results in the Main Text, we take a simpler assumption that the efficacy of surgical masks is at 50%.

Regarding potential concerns on the effectiveness of mask-wearing in reducing the epidemics, we provided additional references and descriptions in the following.

In the laboratory setting, there is evidence that masks are able to filter in the relevant droplet size range for COVID-19, as well as efficacy in blocking droplets and particles from the wearer

in a range higher or near the efficacy of 50% [17, 18]. For seasonal coronaviruses, surgical masks for source control were effective at blocking coronavirus droplets of all sizes for every subject [19]. Personal protection is more challenging than source control, since the inhaling particles are smaller. According to World Health Organisation's "Advice on the use of masks in the context of COVID-19" [20], the penetration for surgical masks is 50%-60% , which is the range we used in our simplified model.

There are already several experimental measurements on the efficacy of different types of masks against coronavirus, both as source control [19, 21, 22] and personal protection equipment [20, 23, 24]. In summary, there is laboratory-based evidence that surgical or N95 masks have satisfying filtration capacity in the relevant droplet size range of coronavirus. The experimental reports are included in the references.

Several recent modelling works focusing on the effect of population-wide mask-wearing converge to similar conclusions that masks of intermediate filtering efficacy exhibit aggregate effect at the population level. For example, in the work of Stutt *et al.* [25], they found that with a policy that all individuals must wear a mask all of the time, a median effective COVID-19 $R_{\mathrm{E}}$ of below 1 could be reached, even with mask effectiveness of 50% (for $R_{\mathrm{E}} = 2.2$) or mask effectiveness of 75% (for $R_{\mathrm{E}} = 4$). Similarly, models from Kai *et al.* [26] estimated that 80-90% masking would eventually eliminate the disease. Work from Fisman *et al.* [27] also showed similar results.

We particularly considered the situation that the filtering efficacy of masks being relatively low, and asked whether masks with intermediate efficacy in personal protection (for example, only filtering 50% of viruses) can have an aggregate effect when applied on a population-wide scale. In Supplementary Figure 4, masks with different filtering efficacy are considered. The results show that even masks only trapping 20% of the virus can bring a significant impact when generally adopted by the population. Also, in the model shown in http://www.zhiyuanlab.xyz/MASK_0906.html, more details related to the effect of population-wide mask-wearing were considered, such as infections relying on non-respiratory routes, and the different filtering efficacies for masks as source control and personal protection.

Last but not least, via comparing cases in German regions with and without masks and weighing various estimates, Mitze *et al.* [28] concluded that 20 days after becoming mandatory face masks had reduced the number of new infections by around 45%, with economic costs close to zero compared to other public health measures.

### 4.3 Solution with imported cases

Border control measures can effectively stop imported cases of viral carriers. To examine the time needed for their effect to take place, let us first consider growth driven by imported cases when unchecked. Under a daily flux $J_{\mathrm{ext}}(T)$ of imported cases into the phase $\mathrm{A}_1$, Eq. (S3) is modified to,

$$A_1(T) = \int_0^\infty \Big(r(t)A_1(T-t) + e^{-\alpha_{\mathrm{A}} t} J_{\mathrm{ext}}(T-t)\Big)dt. \tag{S50}$$

Consider a simple situation where the imported cases grow exponentially with a rate $\lambda$, i.e. $J_{\mathrm{ext}}(T) = J_{\mathrm{I}} e^{\lambda_{\mathrm{I}}(T-T_0)}$. Let's seek an exponentially growing solution where the local population is driven by the imported cases (i.e., the local growth rate in the absence of imported cases is less than $\lambda_{\mathrm{I}}$),

$$\begin{aligned}
A_1(T) &= A_{\mathrm{all}}\, e^{\lambda_{\mathrm{I}}(T-T_0)}, \\
A_{\mathrm{all}} &= \tilde{r}\,(\lambda_{\mathrm{I}})\, A_{\mathrm{all}} + \frac{J_{\mathrm{I}}}{\lambda_{\mathrm{I}} + \alpha_{\mathrm{A}}}, \\
A_{\mathrm{all}} &= \frac{J_{\mathrm{I}}}{[1-\tilde{r}\,(\lambda_{\mathrm{I}})](\lambda_{\mathrm{I}} + \alpha_{\mathrm{A}})}.
\end{aligned}$$

The fraction of local infections after the initial transient is given by,

$$\frac{A_{\rm local}}{A_{\rm all}} = \tilde{r}\left(\lambda_{\rm I}\right). \tag{S51}$$

In many cases, the ratio of imported and local infections is known. Equation (S51) can then be used to calibrate the level of local transmission when the imported cases grow exponentially at a rate greater than the one given in Supplementary Table 2 for local outbreaks.

### 4.4 Crossover behaviour under linear transmission reduction

We consider here a situation where the disease transmission rate per infected individual in a given community gradually weakens according to the schedule,

$$\eta(T) = 1 - (1-\eta_1)\frac{T - T_{\rm i}}{\tau}, \quad T_{\rm i} < T < T_{\rm i} + \tau.$$

Here $T_{\rm i}$ is the starting date of the intervention and $\tau$ is the duration of the crossover period. For $T < T_{\rm i}$ and $T > T_{\rm i} + \tau$, $\eta(T)$ is equal to 1 and $\eta_1$, respectively. For sufficiently slow variation of $\eta(T)$, i.e., $\tau$ is significantly greater than the width of the infectiousness peak which lasts for 2-3 days, we may take the "adiabatic approximation" $R_{\rm E}(T) \simeq \eta(T)R_0$. Equation (S2) then takes the form,

$$A_1(T) = \int_0^\infty r(t)\eta(T-t)A_1(T-t)dt. \tag{S52}$$

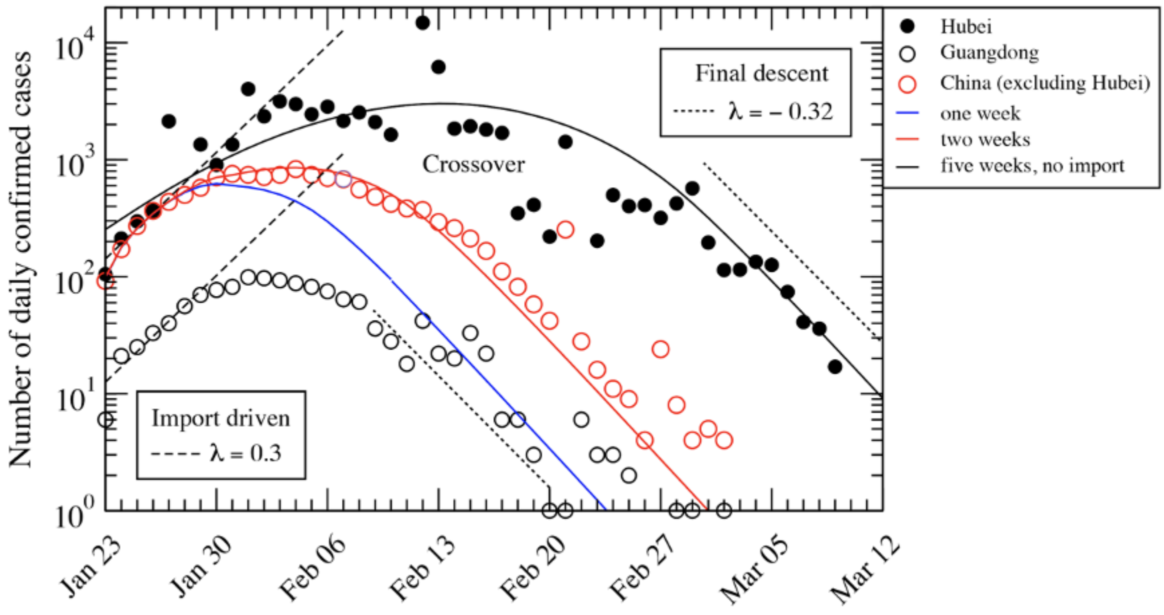


**Supplementary Figure 5: Simulation of the number of daily confirmed cases with a linear decay of transmission per infected individual.** The number of daily confirmed cases in Hubei, Guangdong and China (excluding Hubei) are re-plotted for comparison. Note that the lockdown in Wuhan was started on the date Jan 23. Cluster infections result in occasional bursts in the time series which are not modeled in this work. The time shown in the legend corresponds to the value of $\tau$ in each case.

We used Eq. (S52) to simulate the epidemic development curves in China after the Wuhan lockdown (Main Text Figures 4a and 4b), taking $\eta_1 = 0$. The starting time of the social distancing policy $T_{\rm i}$ is chosen to be one week after the lockdown. The blue, red and black

curves in Supplementary Figure 5 correspond to three different values of $\tau$ given in the legend. The situation represented by blue and red curves is initially driven by imported cases whose number grows exponentially at a rate $\lambda_0 = 0.2$/day prior to the lockdown, with an amplitude that decreases linearly and vanishes on the 10th day after the lockdown. No imported cases were introduced to generate the black curve. Taken at face value, our model can reproduce fast or slow crossovers seen in the data from various provinces of China. On the scale of tens of millions of people, Eq. (S52) can only be considered as representing an aggregated trend helped by the linear nature of the model. As the epidemic spreads into disparate communities, there could be huge variations in the level of transmission from community to community, particularly as the policies tighten. Occasional outbreaks are evident from the scattered data in the declining phase of the pandemic.

### 4.5 Homestay

The situation on the cruise ship Diamond Princess is close to a sudden complete confinement of the passengers. Such a scenario is described by the schedule function

$$\eta(T) = \begin{cases} 1, & T < T_\mathrm{i} \\ \eta_1, & T > T_\mathrm{i} \end{cases}$$

at $\eta_1 = 0$. Under the Markovian approximations introduced in Sec. 3.2, the change over from exponential growth to exponential decay can be solved analytically.

Take $A_1(T) = A_0 e^{\lambda_0 (T - T_\mathrm{i})}$ for $T < T_\mathrm{i}$ , we may rewrite Eq. (S52) as,

$$A_1(T) = \eta_1 \int_0^{T-T_\mathrm{i}} r(t) A_1(T-t) dt + S(T), \tag{S53}$$

where

$$S(T) = \int_{T-T_\mathrm{i}}^{\infty} r(t) A_0 e^{\lambda_0 (T - T_\mathrm{i} - t)} dt.$$

Performing the Laplace transform of Eq. (S53) with

$$\tilde{A}_1(\lambda) = \int_{T_\mathrm{i}}^{\infty} A_1(T) e^{-\lambda (T - T_\mathrm{i})} dT,$$

we obtain,

$$\tilde{A}_1(\lambda) = \frac{\tilde{S}(\lambda)}{1 - \eta_1 \tilde{r}(\lambda)}, \tag{S54}$$

where

$$\tilde{S}(\lambda) = \int_{T_\mathrm{i}}^{\infty} S(T) e^{-\lambda (T - T_\mathrm{i})} dT = A_0 \frac{\tilde{r}(\lambda_0) - \tilde{r}(\lambda)}{\lambda - \lambda_0}.$$

Writing

$$\tilde{A}_1(\lambda) = A_0 \frac{\tilde{r}(\lambda_0)}{\lambda - \lambda_0} \frac{\tilde{r}^{-1}(\lambda) - \tilde{r}^{-1}(\lambda_0)}{\tilde{r}^{-1}(\lambda) - \eta_1}.$$

Under the Markov approximation Eq. (S43), we have

$$\tilde{r}^{-1}(\lambda) \simeq R_\mathrm{E}^{-1} \left(1 + \frac{\lambda}{\alpha_\mathrm{L}}\right) \left(1 + \frac{\lambda}{\alpha_\mathrm{A,eff}}\right).$$

Consequently,

$$\tilde{A}_1(\lambda) = A_0 \tilde{r}(\lambda_0) \frac{\lambda + \lambda_0 + \alpha_\mathrm{L} + \alpha_\mathrm{A,eff}}{(\lambda + \alpha_\mathrm{L})(\lambda + \alpha_\mathrm{A,eff}) - \eta_1 \alpha_\mathrm{L} \alpha_\mathrm{A,eff} R_\mathrm{E}}. \tag{S55}$$

Carrying out the inverse Laplace transform of Eq. (S55), we obtain

$$A_1(T) = A_0 \left( B_+ e^{\lambda_+(T-T_\mathrm{i})} + B_- e^{\lambda_-(T-T_\mathrm{i})} \right). \tag{S56}$$

Here,

$$\lambda_\pm = \frac{-\left(\alpha_\mathrm{L} + \alpha_\mathrm{A,eff}\right) \pm \sqrt{\left(\alpha_\mathrm{L} - \alpha_\mathrm{A,eff}\right)^2 + 4R_1\alpha_\mathrm{L}\alpha_\mathrm{A,eff}}}{2},$$

$$B_+ = \frac{\lambda_+ + \lambda_0 + \alpha_\mathrm{L} + \alpha_\mathrm{A,eff}}{\lambda_+ - \lambda_-},$$

$$B_- = 1 - B_+,$$

with

$$R_1 = \eta_1 R_\mathrm{E} = \eta_1 \frac{(\alpha_\mathrm{L} + \lambda_0)(\alpha_\mathrm{A,eff} + \lambda_0)}{\alpha_\mathrm{L}\alpha_\mathrm{A,eff}}.$$

Note that $\tilde{r}(\lambda_0) = 1$ according to Eq. (S10).

The solution (S56) is shown for several examples in Supplementary Figure 6, where we assumed the "daily confirmed" cases to be proportional to $A_1(T)$. Note that the duration of the crossover is set by $\alpha_\mathrm{L}^{-1} \simeq 3$ days for the parameters chosen. At $\eta_1 = 1$ (no homestay order), we have $\lambda_+ = \lambda_0$ and $B_+ = 1$, as can be verified from the expressions given above.

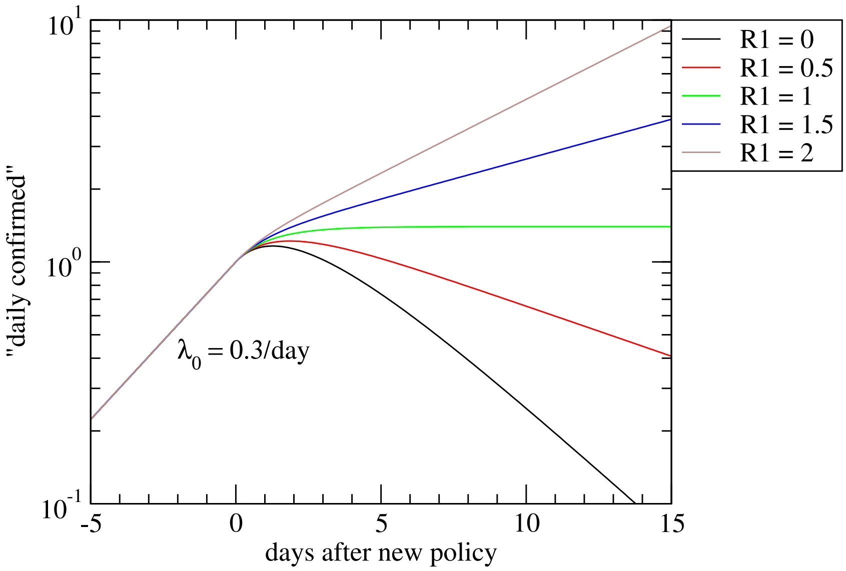


**Supplementary Figure 6: Simulation of daily confirmed infections under a sudden reduction of $R_\mathrm{E}$.** The epidemic initially grows at a rate $\lambda_0 = 0.3$/day before the jump, corresponding to $R_\mathrm{E} = R_0 = 3.8$. Different curves correspond to different values of $R_1 = \eta_1 R_0$ under the new policy that starts on day $T = T_\mathrm{i}$. Here $\alpha_\mathrm{L} = 0.3$/day, $\alpha_\mathrm{A,eff} = 0.5$/day.

## 5 Estimation of exponential growth rates during initial outbreaks

The reported COVID-19 infection cases in various countries and regions typically show exponential growth with time during the initial outbreak of the disease. The growth rate $\lambda$ is of great interest not only to epidemiologists, but also to the general public. In theory, $\lambda$ can be determined by fitting the cumulative number of confirmed cases to an exponential function of time (days). However, when the number of cases is small, growth tends to be affected by chance events (e.g., super-spreaders) and also by family cases where the transmission pattern can be quite different from that of typical social contacts. Furthermore, it may be dominated by imported cases which do not reflect much of the spreading characteristics in the local community. Therefore caution is required in using the data to extract the epidemic growth rate which itself may vary with time due to the mitigation and intervention measures introduced.

In their study of epidemic data for dengue fever in Brazil, Favier *et al.* [29] proposed to plot the daily new cases $\Delta N$ against the cumulative confirmed cases $N(T)$ and to fit the data with a linear function to determine $\lambda$. For perfect exponential growth, we have

$$\frac{\Delta N(T)}{N(T)} \equiv \frac{N(T) - N(T - \Delta T)}{N(T)} = 1 - \exp(-\lambda \Delta T), \tag{S57}$$

where $\Delta T = 1$ day. Therefore the slope of the scatter plot $\Delta N(T)$ against $N(T)$ can be used to estimate $\lambda$. The next step is to determine the interval over which the fitting is carried out.

Following the work by Favier *et al.*, we performed linear fits of $\Delta N(T)$ against $N(T)$, each time using one more data point, through which we obtain the slope and goodness-of-fit of these linear regressions as a function of cumulative number of confirmed cases. Then considering the 80th percentile of the slopes left to the minimum goodness-of-fit with goodness-of-fit smaller than 0.5 as the exponential phase, we obtain an estimate of the growth rate. The data and fitted linear functions are shown in Supplementary Figure 7.

In Supplementary Table 10, we collect the estimated growth rates using the above scheme, along with the statistics of the linear regression (R-squared and p-value for the F-test) and also the total case number in the exponential growth phase for different countries/regions. Where applicable, the estimated values are in good agreement with the location of the plateaus in Main Text Figure 6d. Given the many technical issues involved, we prefer not to include this particular analysis in the Main Text but instead have made revisions to the effect that the epidemic growth rate generally varied from place to place.

Solving for $\lambda$ in Eq. (S57), we obtain,

$$\lambda(T) = \frac{\ln N(T) - \ln N(T - \Delta T)}{\Delta T}. \tag{S58}$$

Therefore we may also think of the above procedure as computing the instantaneous growth rates from the $\ln N(T)$ against $T$ curve, with $\Delta T$ chosen suitably to minimize irregularities in testing and reporting. Given the mean incubation period of about 6 days for COVID-19, $\lambda(T)$ is expected to be a slow-varying quantity with a typical timescale of a week or longer. Five examples of $\lambda(T)$ versus $N(T)$ during the initial local outbreaks are given in Main Text Figure 6d of the Main Text. Reasonably good agreement is seen between the plateau value of $\lambda(T)$ and the growth rates listed in Supplementary Table 10 in four of the five countries, with the exception of Italy, where the substantially lower value of 0.26/day in the table corresponds to the second plateau shown in Main Text Figure 6d.

Provincial epidemic growth in China after the Wuhan lockdown has a large component of imported cases. The epidemic development in the Hubei province, on the other hand, is subject to swift intervention and containment measures such as isolation of patients in Fangcang hospitals [30]. The situation in South Korea is also special due to a well-documented outbreak in Daegu in the second half of February [31]. Therefore the growth rates listed in Supplementary Table 10 in these locations need to be interpreted differently.

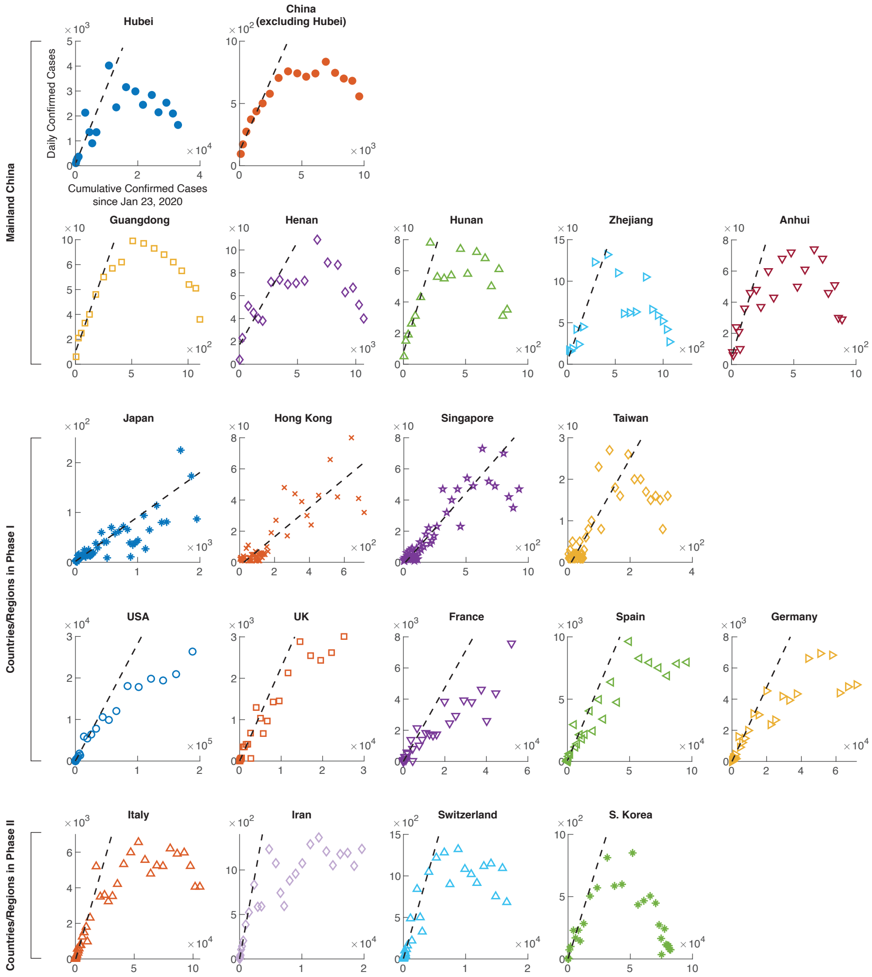


**Supplementary Figure 7:** Linear regression of the number of daily confirmed cases as a function of the cumulative number of confirmed cases in the beginning of the COVID-19 pandemic in selected countries/regions.

**Supplementary Table 10:** Estimated exponential growth rate $\lambda$, linear regression statistics (coefficient of determination $R^2$ and $p$-value for the one-sided $F$-test), and total case number in exponential growth phase since January 23, 2020 for different countries/regions, following the scheme of Favier *et al.* [29]. The calculated mean reproduction number $R_E$ is also shown (95% confidence interval shown in parentheses).

| Countries /Regions | $\lambda$ | $R^2$ | $p$-value ($F$-test) | Total case number in exponential phase | Mean reproduction number $R_E$ |
|---|---|---|---|---|---|
| Hubei | 0.3661 | 0.7678 | 0.0019 | 10,733 | 4.94 (4.21,5.89) |
| China (excluding Hubei) | 0.2571 | 0.9443 | 0.0012 | 1,851 | 3.28 (2.93,3.71) |
| Guangdong | 0.2975 | 0.9620 | 5.4775e-04 | 181 | 3.83 (3.37,4.42) |

| | | | | | |
|---|---|---|---|---|---|
| Henan | 0.1999 | 0.6634 | 0.0257 | 273 | 2.60 (2.38,2.87) |
| Hunan | 0.3004 | 0.9697 | 3.4891e-04 | 139 | 3.88 (3.40,4.48) |
| Zhejiang | 0.3985 | 0.9165 | 1.8789e-04 | 418 | 5.53 (4.64,6.70) |
| Anhui | 0.3079 | 0.7994 | 0.0066 | 151 | 3.99 (3.49,4.63) |
| Japan | 0.0934 | 0.7243 | 1.3134e-09 | 418 | 1.62 (1.55,1.70) |
| Hong Kong | 0.0975 | 0.6664 | 5.3861e-14 | 410 | 1.65 (1.58,1.73) |
| Singapore | 0.0960 | 0.7422 | 3.0786e-14 | 345 | 1.64 (1.57,1.72) |
| Taiwan | 0.1416 | 0.6959 | 4.2117e-11 | 168 | 2.02 (1.90,2.17) |
| USA | 0.3318 | 0.9398 | 4.2590e-21 | 25,488 | 4.36 (3.77,5.12) |
| UK | 0.2561 | 0.8222 | 7.2719e-11 | 5,018 | 3.27 (2.92,3.69) |
| France | 0.2701 | 0.7119 | 3.2394e-08 | 6,633 | 3.45 (3.07,3.93) |
| Spain | 0.2691 | 0.7469 | 2.1631e-07 | 9,942 | 3.44 (3.06,3.91) |
| Germany | 0.2715 | 0.8255 | 9.6580e-12 | 5,795 | 3.47 (3.08,3.95) |
| Italy | 0.2575 | 0.8755 | 5.7844e-11 | 17,660 | 3.29 (2.94,3.72) |
| Iran | 0.4821 | 0.9937 | 2.4192e-12 | 978 | 7.34 (5.91,9.21) |
| Switzerland | 0.3019 | 0.8068 | 4.1837e-07 | 2,700 | 3.90 (3.42,4.51) |
| S. Korea | 0.3858 | 0.8871 | 1.9155e-10 | 832 | 5.29 (4.47,6.37) |

# References


[1] Kermack, W.O. & McKendrick, A.G. A contribution to the mathematical theory of epidemics. *Proc R Soc Lond A* **115**, 700-721 (1927).

[2] Diekmann, Q., Heesterbeek, J. A. P. & Metz, J. A. J. On the definition and the computation of the basic reproduction ratio $R_0$ in models for infectious diseases in heterogeneous populations. *J Math Biol* **28**, 365-382 (1990).

[3] Heesterbeek, J. A. P. & Dietz, K. The concept of $R_0$ in epidemic theory. *Statistica Neerlandica* **50**, 89-110 (1996).

[4] Breda, D. *et al.* On the formulation of epidemic models (an appraisal of Kermack and McKendrick). *J Biol Dyn.* **6**, sup2, 103-117 (2012).

[5] Wallinga, J. & Lipsitch, M. How generation intervals shape the relationship between growth rates and reproductive numbers. *Proc R Soc B Biol Sci.* **274**, 599-604 (2007).

[6] Men, K. *et al.* Estimate the incubation period of coronavirus 2019 (COVID-19). *medRxiv*:2020.02.24.20027474 (2020). https://doi.org/10.1101/2020.02.24.20027474

[7] Xia, W. *et al.* Transmission of corona virus disease 2019 during the incubation period may lead to a quarantine loophole. *medRxiv*:2020.03.06.20031955 (2020). https://doi.org/10.1101/2020.03.06.20031955

[8] Bi, Q. *et al.* Epidemiology and transmission of COVID-19 in 391 cases and 1286 of their close contacts in Shenzhen, China: a retrospective cohort study *Lancet Infect Dis.* **20**, 911-919 (2020).

[9] Reich, N.G. *et al.* Estimating incubation period distributions with coarse data. *Stat Med.* **28**, 2769-2784 (2009).

[10] Du, Z. *et al.* Serial Interval of COVID-19 among Publicly Reported Confirmed Cases *Emerg Infect Dis.* **26**, 1341-1343 (2020).

[11] Xu, X.K. *et al.* Reconstruction of transmission pairs for novel coronavirus disease 2019 (COVID-19) in mainland China: estimation of super-spreading events, serial interval, and hazard of infection *Clin Infect Dis.*, ciaa790 (2020). https://doi.org/10.1093/cid/ciaa790

[12] He, X. et al. Temporal dynamics in viral shedding and transmissibility of COVID-19. *Nat Med.* **26**, 672-675 (2020).

[13] Ali, S.T. *et al.* Serial interval of SARS-CoV-2 was shortened over time by nonpharmaceutical interventions. *Science* **369**, 1106-1109 (2020).

[14] Milton, D.K. *et al.* Influenza virus aerosols in human exhaled breath: particle size, culturability, and effect of surgical masks. *PLoS Pathog.* **9**, e1003205 (2013).

[15] World Health Organization. *Modes of transmission of virus causing COVID-19: implications for IPC precaution recommendations.* Available at: https://www.who.int/newsroom/commentaries/detail/modes-of-transmission-of-virus-causing-covid-19-implications-for-ipc-precaution-recommendations (Accessed: 2nd April 2020).

[16] ASTM F2101-19, Standard Test Method for Evaluating the Bacterial Filtration Efficiency (BFE) of Medical Face Mask Materials, Using a Biological Aerosol of Staphylococcus aureus, ASTM International, West Conshohocken, PA, 2019. http://www.astm.org/cgi-bin/resolver.cgi?F2101

[17] Prather, K.A., Wang, C.C. & Schooley, R.T. Reducing transmission of SARS-COV-2. *Science* **368**, 1422-1424 (2020).

[18] Gralton, J. *et al.* The role of particle size in aerosolised pathogen transmission: a review. *J Infect.* **62**, 1-13 (2011).

[19] Leung, N.H. *et al.* Respiratory virus shedding in exhaled breath and efficacy of face masks. *Nat Med.* **26**, 676-680 (2020).

[20] World Health Organization. *Advice on the use of masks in the context of COVID-19 – interim guidance.* Available at: https://www.who.int/publications/i/item/advice-on-the-use-of-masks-in-the-community-during-home-care-and-in-healthcare-settings-in-the-context-of-the-novel-coronavirus-(2019-ncov)-outbreak (Accessed: 2nd April 2020).

[21] Chan, J.F.-W. *et al.* Surgical mask partition reduces the risk of non-contact transmission in a golden syrian hamster model for coronavirus disease 2019 (covid-19). *Clin Infect Dis.*, ciaa644 (2020). https://doi.org/10.1093/cid/ciaa644

[22] Viola I.M. *et al.* Face coverings, aerosol dispersion and mitigation of virus transmission risk. *arXiv*:2005.10720 (2020). https://arxiv.org/abs/2005.10720

[23] Jung, H. *et al.* Comparison of filtration efficiency and pressure drop in anti-yellow sand masks, quarantine masks, medical masks, general masks, and handkerchiefs. *Aerosol Air Qual Res.* **14**, 991-1002 (2014).

[24] Verma, S., Dhanak, M. & Frankenfield, J. Visualizing the effectiveness of face masks in obstructing respiratory jets. *Phys Fluids* **32**, 061708 (2020).

[25] Stutt, R.O. *et al.* Modelling framework to assess the likely effectiveness of facemasks in combination with lock-down in managing the covid-19 pandemic. *Proc Royal Soc A* **476**, 20200376 (2020).

[26] Kai, D. *et al.* Universal masking is urgent in the covid-19 pandemic: SEIR and agent based models, empirical validation, policy recommendations. *arXiv*:2004.13553 (2020). https://arxiv.org/abs/2004.13553

[27] Fisman, D.N., Greer, A.L. & Tuite, A.R. Brief research report: Bidirectional impact of imperfect mask use on reproduction number of covid-19: A next generation matrix approach. *Infect Dis Model.* **5**, 405-408 (2020).

[28] Mitze, T., Kosfeld, R., Rode, J. & Wälde, K. Face masks considerably reduce COVID-19 cases in Germany. *Proc Natl Acad Sci U S A* **117**, 32293-32301 (2020).

[29] Favier, C. *et al.* Early determination of the reproductive number for vector-borne diseases: the case of dengue in Brazil. *Trop Med Int Health* **5**, 332-340 (2006).

[30] Chen S. *et al.* Fangcang shelter hospitals: a novel concept for responding to public health emergencies. *Lancet* **395**, 1305–14 (2020).

[31] Shim E. *et al.* Transmission potential and severity of COVID-19 in South Korea. *Int J Infect Dis.* **93**, 339–344 (2020).
