# Supplementary material for: Harnessing peak transmission around symptom onset for non-pharmaceutical intervention and containment of the COVID-19 pandemic
Source: Nat Commun. 2021 Feb 19;12:1147. doi: 10.1038/s41467-021-21385-z (PMC7895830; doi:10.1038/s41467-021-21385-z)
Supplement: Supplementary file 3 — Reporting Summary [file 41467_2021_21385_MOESM3_ESM.pdf]

nature research

Corresponding author(s): Lei-Han Tang

Last updated by author(s): Jan 11, 2021

Reporting Summary

Nature Research wishes to improve the reproducibility of the work that we publish. This form provides structure for consistency and transparency in reporting. For further information on Nature Research policies, see our Editorial Policies and the Editorial Policy Checklist.

Statistics

For all statistical analyses, confirm that the following items are present in the figure legend, table legend, main text, or Methods section.

n/a	Confirmed	
☐	☒	The exact sample size (n) for each experimental group/condition, given as a discrete number and unit of measurement
☐	☒	A statement on whether measurements were taken from distinct samples or whether the same sample was measured repeatedly
☐	☒	The statistical test(s) used AND whether they are one- or two-sided<br>*Only common tests should be described solely by name; describe more complex techniques in the Methods section.*
☒	☐	A description of all covariates tested
☐	☒	A description of any assumptions or corrections, such as tests of normality and adjustment for multiple comparisons
☐	☒	A full description of the statistical parameters including central tendency (e.g. means) or other basic estimates (e.g. regression coefficient) AND variation (e.g. standard deviation) or associated estimates of uncertainty (e.g. confidence intervals)
☐	☒	For null hypothesis testing, the test statistic (e.g. F, t, r) with confidence intervals, effect sizes, degrees of freedom and P value noted<br>*Give P values as exact values whenever suitable.*
☒	☐	For Bayesian analysis, information on the choice of priors and Markov chain Monte Carlo settings
☒	☐	For hierarchical and complex designs, identification of the appropriate level for tests and full reporting of outcomes
☒	☐	Estimates of effect sizes (e.g. Cohen's d, Pearson's r), indicating how they were calculated

Our web collection on statistics for biologists contains articles on many of the points above.

Software and code

Policy information about availability of computer code

Data collection	No software was used for data collection. All the COVID-19 related data analyzed in this work, such as confirmed case number, incubation period, exposure window of transmission pair, and serial interval, are from published studies or datasets.
Data analysis	All the data analysis was performed in MATLAB R2016b. Our code can be downloaded here: https://github.com/hkbu-covid19group/Calib-covid19

For manuscripts utilizing custom algorithms or software that are central to the research but not yet described in published literature, software must be made available to editors and reviewers. We strongly encourage code deposition in a community repository (e.g. GitHub). See the Nature Research guidelines for submitting code & software for further information.

Data

Policy information about availability of data

All manuscripts must include a data availability statement. This statement should provide the following information, where applicable:

- Accession codes, unique identifiers, or web links for publicly available datasets
- A list of figures that have associated raw data
- A description of any restrictions on data availability

The COVID-19 pandemic and clinical data used in this work are all from published studies or datasets: confirmed case number (COVID-19 Data Repository by the Center for Systems Science and Engineering at Johns Hopkins University: https://github.com/CSSEGISandData/COVID-19), incubation period (three datasets from Refs. 11, 20, and 21), exposure window of transmission pair (one dataset from Ref. 7), and serial interval (one dataset from Refs. 22 and 23). Data for the Diamond Princess cruise ship are from Ref. 13. These datasets are included in Sourcedata for easy reference, together with the parametrised distributions and the R_E-λ relation from this work.

Field-specific reporting

Please select the one below that is the best fit for your research. If you are not sure, read the appropriate sections before making your selection.

- [x] Life sciences
- [ ] Behavioural & social sciences
- [ ] Ecological, evolutionary & environmental sciences

For a reference copy of the document with all sections, see nature.com/documents/nr-reporting-summary-flat.pdf

Life sciences study design

All studies must disclose on these points even when the disclosure is negative.

Sample size	The COVID-19 related data analyzed in this work, such as confirmed case number, incubation period, exposure window of transmission pair, and serial interval, are all from published studies or datasets. The data collection protocols can be found in original publications. Incubation period: 105 cases from Ref. 11; 59 cases from Ref. 20; 183 cases from Ref. 21. Exposure window statistics of transmission pairs: 66 out of 77 transmission pairs with clear infector symptom onset date from Ref. 7 were used. Serial interval: 677 out of 1407 transmission pairs with clear symptom onset dates and social relationships of infector-infectees from Ref. 22 were used.
Data exclusions	Exposure window statistics of transmission pairs: 11 out of 77 transmission pairs without infector symptom onset date in Ref. 7 were excluded. Serial interval: 730 out of 1407 transmission pairs without symptom onset dates and social relationships of infector-infectees in Ref. 22 were excluded. The exclusion is purely due to the incomplete sample information, which was also adopted in other studies, such as Ref. 23.
Replication	Three independent datasets of incubation period were used for parameter calibration. Another independent dataset of serial interval was used to cross-validate our estimated parameters.
Randomization	Randomization and sample allocation were not involved in this study.
Blinding	Blinding was not involved in this study.

Reporting for specific materials, systems and methods

We require information from authors about some types of materials, experimental systems and methods used in many studies. Here, indicate whether each material, system or method listed is relevant to your study. If you are not sure if a list item applies to your research, read the appropriate section before selecting a response.

Materials & experimental systems

n/a	Involved in the study	
x		Antibodies
x		Eukaryotic cell lines
x		Palaeontology and archaeology
x		Animals and other organisms
x		Human research participants
x		Clinical data
x		Dual use research of concern

Methods

n/a	Involved in the study	
x		ChIP-seq
x		Flow cytometry
x		MRI-based neuroimaging
